# Life satisfaction in families with a child in an Unresponsive Wakefulness Syndrome

**DOI:** 10.1186/s12887-021-02549-8

**Published:** 2021-03-08

**Authors:** Christiane Chadasch, Boris Kotchoubey

**Affiliations:** 1 Management Psychology, Coping, Resilience & Ethics, Meiersheide 8a, 53773 Hennef, Germany; 2 Institute of Medical Psychology and Behavioral Neurobiology, Eberhardt Karls University of Tübingen, Silcherstr. 5, 72076 Tübingen, Germany

**Keywords:** Unresponsive wakefulness syndrome (UWS), Coping, Resilience, Self-management, Children

## Abstract

**Background:**

The article examines life conditions in families living together with a child in an Unresponsive Wakefulness Syndrome (UWS). Such families experience severe stress at financial, logistical, and existential level.

**Methods:**

We investigated a large sample of families living with a UWS child (comprising 13% of the total population) and compared these families with families without a chronically ill child. A set of four questionnaires aimed to evaluate life conditions entails a total of 204 items. One of the questionnaires was developed by the corresponding author specifically for this study. The questionnaires were positively accepted by the persons concerned and permitted us to test six specific hypotheses.

**Results:**

Life satisfaction (LS) in families with a UWS child was significantly lower than in control families. LS was significantly affected by external situational factors (everyday support, home visits, support by a doctor, nursing service, health insurance, etc.). Self-management skills were on average lower in families with a UWS child than in controls. These skills strongly and directly correlated with LS. Further, LS was not significantly related to the acceptance of feelings and negatively correlated with the floods of emotions. The relationship with the own child was equally satisfactory in families with and without a UWS child indicating that the families regard their UWS child as a full family member.

**Conclusions:**

The data show that happy life is possible in families living together with a UWS child. They further specify conditions for satisfactory life under multiple highly severe challenges. Personal self-management skills, coping strategies, and resilience, as well as outside social support, appear to be critical factors.

**Supplementary Information:**

The online version contains supplementary material available at 10.1186/s12887-021-02549-8.

## Background

Unresponsive Wakefulness Syndrome (UWS; previously referred to as vegetative state) is a condition following severe brain injuries, in which a patient appears to be awake (open eyes, normal or close-to-normal reflexes to simple stimuli), but does not show any sign of conscious awareness or intention to communicate with his/her environment [34, 39]. Although there is no exact statistics for UWS, a systematic review [67] found that its total (adults and children) prevalence broadly vary among different countries from 0.2 per 100,000 population in the Netherlands to 6.1 per 100,000 in Italy. Moreover, while the exceptionally low Dutch estimate (0.2: [40]) was obtained on the basis of studying nursing homes only, a later Dutch study encompassing nursing homes, hospitals, rehabilitation centers, and hospices, revealed an even lower estimate close to 0.15 [68]. These differences can probably be attributed to different thresholds existing in different countries for termination of life-sustaining measures in UWS or in coma preceding UWS [69]. As regards children, more than 25 years ago Ashwal et al. [3] estimated the prevalence of UWS (vegetative state) as 0.63 per 100,000 but supposed in their discussion that the real number must be somewhat higher. This is in line with the later data of Geremek [17], according to which there should be some 600 UWS children in Germany.

About 50% of adult UWS patients later regain consciousness (e.g., [10, 27, 35, 36]). This percentage appears to be higher among UWS children (e.g., [22, 32]). The level of the remaining disability after regaining consciousness varies widely, and the factors that determine this level are still to be explored. Everything else being equal, younger patients have better chances to restore their functions than older patients. However, even in children UWS can be not only a transient but also a chronic or even permanent condition. In many such cases UWS children live at home together with their families, resulting in considerable burden. The present study examines whether a contented life is possible for the families and which factors influence the life situation.

The presence of a UWS child is a severe stress frequently leading to changes in all life planning. The necessity of 24 h care for a completely disabled child results in high pressure that encompasses financial, time-related, organizational, and logistical issues (for similar conditions, see [51, 54]). Several aspects of this multiple stress and ways of coping in families with severely ill and severely disabled children have been analyzed in the literature (e.g., [4, 20, 63]) and discussed in mass media (e.g., [25]), however without a direct connection with the particularly disabled state of UWS.

Beavers [7] carried out a longitudinal study that covered a period of five years and included 157 families of mentally retarded children. Although they cannot be directly compared with children in the current study (mental deficits of children in Beavers’ [7] study was far less severe than in UWS), his data can be of interest here. He came to the conclusion that “competent” families develop a coping pattern making their life together with severely ill children possible. The pattern includes clear understanding of the disability, structural adjustment of family roles, family interaction, balance of needs, self-reflection, self-esteem, and problem-solving orientation.

Retzlaff [55] provided a summary of the data of Beavers [7], Patterson [50], Scorgie et al. [63], Li-Tsang et al. [41] and Yau and Li-Tsang [71] devoted to the process of family adaptation caused by severe disability of their children. The resulting model includes such factors as the life cycle of the family, the quality of family interaction, and previous experiences of the family.

While the challenges experienced by families with severely ill children and their ways of dealing with such challenges are usually described in terms of stress and coping (Lazarus and Folkman), several authors have questioned whether these concepts are most appropriate and exhaustively depict the nature of the problem (e.g., [12, 48]). Other behavioral patterns, related to but not identical with the traditional patterns of coping, may more adequately describe the strategies used by families. One of the alternatives is an approach based on the concept of self-management (e.g., [9, 28]). This concept was primarily developed as an educational approach for patients [31, 43], intended as an opposition to the traditional active expert/passive patients approach and entailing five principal mechanisms: self-directed care, illness management and recovery, shared decision-making, joint crisis planning and wellness planning [31]. Later on, the principles of self-management were applied to patients’ caregivers, parents and family members of chronically ill children (e.g., [6, 59]).

The aim of the present study was to obtain information of whether and, if appropriate, how a contented life is possible for the families affected by, and living together with, the UWS of their child, which problems this particular life constellation poses and which external circumstances support life satisfaction. The background of the study was the idea that families living together with their child in a UWS should get the opportunity to find help they need to live a successful life. When selecting and designing the study, particular attention was paid to the living conditions of families affected by the UWS of their child. A combination of adequate questionnaires was developed gradually, during a long period of informal contacts with the concerned population. The combination of externally constructed questionnaires and the compilation of a separate questionnaire, which focuses in particular on the anamnestic situation of the families affected by UWS, was accepted by the persons concerned.

Thereby six hypotheses were checked. Particularly, we hypothesized that general life satisfaction (LS) is lower in families with a UWS child than in families with healthy children (H1); that, in the families with a UWS child, LS is inversely related to the burden of the family (H2) and to the subjectively experienced emotional load (H3) and directly related to self-management skills (H4) and the ability to accept one’s own (negative) emotions (H5). Finally, we hypothesized that the relationship with the UWS child is worse than the relationship with a healthy child in control families (H6).

## Methods

### Participants

The two main criteria for inclusion into the study were (i) a child with a diagnosis UWS (ICD 10 G 93.80) (synonyms “apallic syndrome”, “vegetative state”, “Wachkoma” or “coma vigile”) and (ii) the family that was living with the child together in the home environment. The exclusion criterion was the age above 18 at the time of the event causing the UWS. Because the respective population is relatively small, in the acquisition phase we contacted families in three German-speaking countries (Germany, Austria and Switzerland). The contacts were initiated through foundations, specialist newspapers, acute hospitals, rehabilitation clinics, self-help groups, hospices, private practices, physiotherapists, intensive care staff, pediatric nursing services, children’s networks, special schools, federal associations, trade fairs, and family members of known UWS patients. The very intensive search resulted in a total of one hundred ninety families corresponding to the above inclusion criteria.

One hundred ninety questionnaires were sent to interested parties, to foundations etc., who passed on them to the families. Thirty questionnaire packages consisting of a cover letter, questionnaires (see below) and an addressed and revised return envelope were answered. Four of them had to be excluded from the study because, contrary to original statements, it was found that the UWS patients were older than 18 at the time point of the event leading to the UWS. Thus the data basis of the present study entails twenty-six UWS families, or 13.7% of the identified population.

To create a control group, one hundred twenty-three randomly chosen families in Germany, Austria and Switzerland, living in the home environment with a healthy child below the age of 24, were contacted. A total of 80 questionnaire packages were sent to families, fifty-two of whom were answered, returned anonymously and correctly. Of these 52, a random selection of twenty-six was used in this study.

The conditions of comparability of the two groups were the similar age of the children (UWS and healthy children, respectively), the similar age of the parents who filled in our questionnaires, and that all families were continuously living together.

The acceptable alpha error (α) was set to .05. Assuming a correlation (r) of .50 and the presence of both within- and between-subject interactions, a test strength analysis using the program G * Power 3.0 [16] results in an optimal sample size of *N* = 52.

### General design

Many families living together with a UWS child are overburdened and often hypersensitized toward the interests of third parties in their case. Some of them experience strong mistrust against experimental studies seeing themselves and their patients rather as objects to obtain some abstract knowledge form which they do not receive any benefit. They are ruled, perhaps unconsciously and intuitively, by the principle [29] that it should be unethical to use some (ill) human persons just as means to attain some other (even good) end. Recent studies indicate how important is taking the motivational state of disabled children’s mothers in building trustful communication with them [52]. The lack of taking into consideration this specific motivational state may result in “study aversion”, and one may even suppose that this aversion is a cause for the present scarcity of knowledge in the domain.

This was one of the reasons to apply a non-experimental approach. The use of questionnaires in the present study was expected to increase participants’ compliance. When selecting and specifying the design, particular attention was paid to the living conditions of families affected by the UWS of their child. Informal conversations with such families gradually led to the development of a set of adequate questionnaires including both externally constructed tools and an original questionnaire, which focuses in particular on the anamnestic situation of the families affected by UWS.

The first of the hypotheses formulated above requires a comparison between the families living with children with and without UWS. To examine the other hypotheses, further distinctions had to be made within the group of families affected by the UWS of their children. The aim of this analysis was to find out whether families with a higher level of life satisfaction have specific personal abilities that contribute to managing this particular life situation as compared with families with lower satisfaction, and whether there is any link between life satisfaction, the starting point, and the individual characteristics and relationship with one’s own children.

### Questionnaires

The assignment of manifest variables (indicators) to theoretical terms is presented below. Life satisfaction is a multidimensional construct, which leads to numerous possibilities of operationalization. To test Hypothesis 1, we selected a high-quality tool broadly used in the German language area. The questionnaire on life satisfaction (FLZ) of Fahrenberg et al. [15] includes individual assessment of global and area-specific life satisfaction, living conditions, and future perspectives. It entails ten scales: health, job and occupation, financial situation, leisure, partnership, relationship with one’s own children, one’s own person, sexuality, friends and relatives, and apartment, as well as a total value. Because many representatives of the presently investigated population live in atypical conditions regarding their work, partnership, and their relationship with their children, the three corresponding scales were excluded from the calculation of general life satisfaction. FLZ does not include areas of social attitudes such as satisfaction with politics, institutions, parties, churches and so on.

Possible positive (supportive) or negative (lack of support) effects of the initial conditions and life situation on the general life satisfaction were investigated to test Hypothesis 2. For this sake the first author developed a questionnaire on everyday family life with a UWS child (FFCv: [11]). The development was originally based on the long-term experience of social and pedagogical contact with affected families and nursing personnel. Due to this primary experience of the cooperation with UWS families, the questionnaire was able to cover the needs of the child in the home environment, the assistance given to the affected family, and the conditions for community life. At the same time, it satisfied the criteria of good compliance, comprehensibility, clarity and limited scope of work. The semantic and pragmatic understanding of the questions was checked in the pretest.

The questionnaire [11] includes both nominal and ordinal scales, as well as both dichotomous and continuous variables. The answers presented as continuous variables were converted into binary variables referred to as positive (supportive) or negative (deficient) conditions [11]. Thus, the help during daytime and nighttime were rated as positive when it took at least one hour (per 12 h), and as deficient in the opposite case. Low age was considered “positive” because the care for small children does not differ very much between children with and without UWS. In contrast, higher age was considered “negative” because an older UWS child presents a much larger burden for the family as compared with healthy children of the same age. Regarding the support of the nursing service, the social pediatric center, and the insurances, a yes-no-coding define the category “positive” or “deficient”.

The Resource and Self-Management Capability Questionnaire (FERUS) focuses on a person’s supportive abilities [26] and served to validate Hypothesis 4. The theoretical basis of FERUS includes such concepts as salutogenesis [1, 2], self-management [28], self-efficacy [5] and social support [64]. The questionnaire contains seven scales (coping, self-monitoring, self-efficiency, self-verbalization, hope, change motivation, and social support). However, on the basis of his factor analysis Jack [26] recommended to calculate the total FERUS scale without the last two scales, and we followed this recommendation.

The scales for experiencing emotions (SEE) [8] were used to test Hypotheses 3 and 5. The theoretical basis for the formulation of items was the person-centered personality theory [56] and the concept of emotional intelligence [60].

To test Hypothesis 6, we used the scale for *relationship with own children* from the questionnaire of life satisfaction (FLZ) described above.

English translations of all items are given in Supplement 1.

## Results

### General characteristic of the families

90.4% of the respondents lived in Germany and the remaining 9.6% in Austria. 92.3% of the responders were females. Their mean age was 42.6 years (42.7 and 42.5, for the affected group and the control group, respectively) with a range of 29–61 years. The difference between the groups was not significant (t = 0.11; df = 50; *p* = 0.86).

The mean age of the children was 10.96 years (11.12 years in the affected, 10.82 years in the control group) with a range of 2–24 years. The difference between the groups was not significant (t = − 0.10; df = 50; *p* = 0.92).

The mean age of UWS children at the time point of the event was 4.11 years (standard deviation = 5.0, median = 2.5; range between 0 and 17); in 50% of the children it was one of the first two years of life. UWS was caused by traumatic (30% of the cases) or non-traumatic brain injury (61.5%), brain diseases (26.9%), birth trauma (3.8%) or other events (7.7%). Nine of the 26 UWS children had carried a tracheostomy tube and four had been ventilated at least for some time during the illness. 92.3% of UWS children were fed by means of percutaneous endoscopic gastrostomy. 80.8% of UWS children could visit a kindergarten or school (for short time, accompanied by a nurse), which time could be used by the parents to have a break and to stay alone at home.

Fourteen respondents in the UWS group but only 7 respondents in the control group were unemployed (χ^2^ = 3.91, df = 1, *p* = .048). As shown in Table 1, education level was slightly higher in families with UWS children, as compared with the families with healthy children, but the difference was not significant (χ^2^ = 1.34, df = 2, *p* > .5).

**Table 1:** Education level in affected and control families

School education	UWS families		Control families	
	Frequency	Percentage	Frequency	Percentage
Basic school	6	23,1%	7	26,9%
Middle-level school („Realschule“)	6	23,1%	9	34,6%
High school	14	53,8%	10	38,5%

The occupational groups of the respondents showed a similar distribution. The parents of the UWS affected children were more frequently freelancers, whereas parents in the control group more often held senior positions. Both parties were almost equally represented in the non-executive functions. The range of help varied from no help at all to 24 h per day. The rate of emergency situations ranged from zero (15.4%) to four times a year (23.1%). 92.3% received help from medical experts.

### Life satisfaction in UWS families

**Fig. 1:**
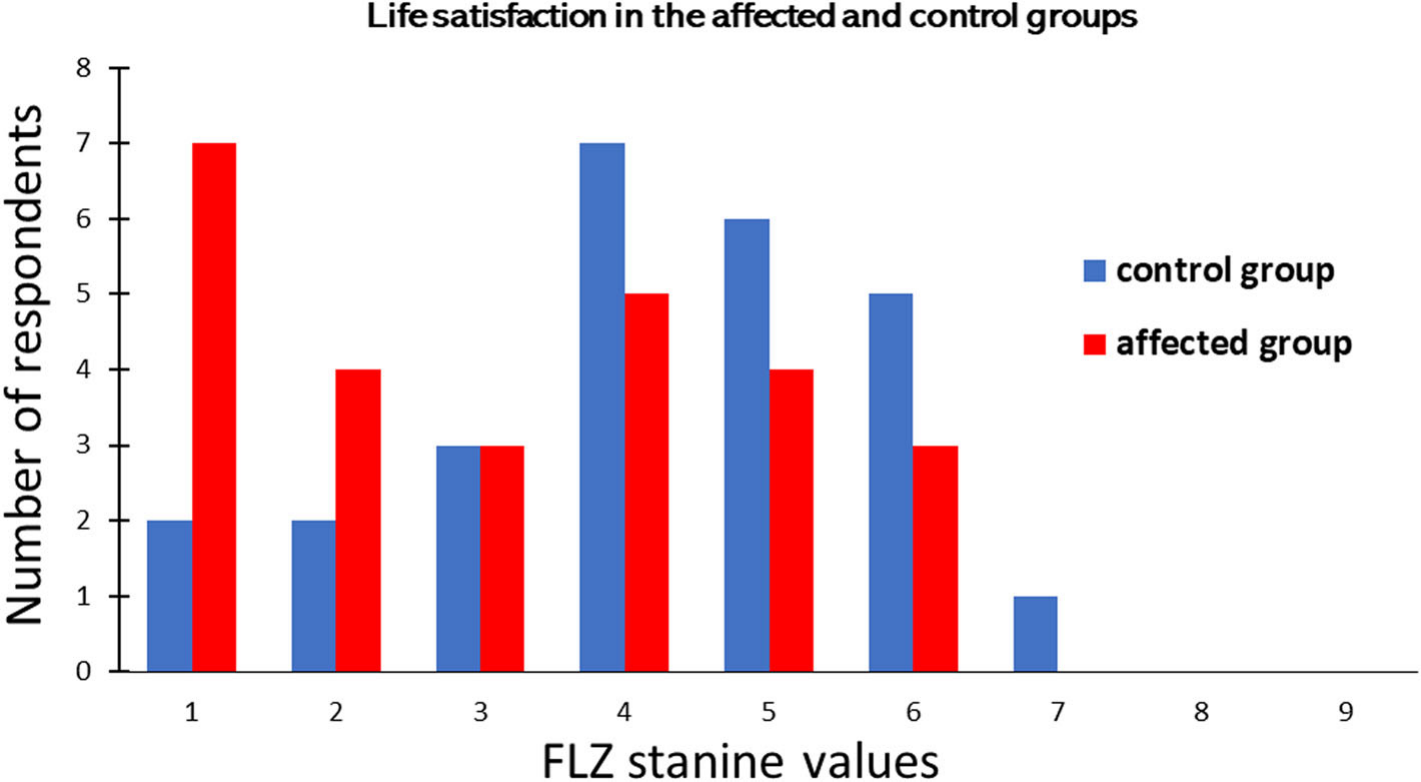
Distribution of Life Satisfaction total scores (standardized FLZ values) in the UWS-affected and control groups. One can see that average satisfaction values (stanines 4 to 6) are more frequent in control families (18 respondents, i.e., 69% of the sample) than in families with a UWS child (12 respondents, i.e., 46%). In contrast, low satisfaction values (stanines 1 and 2) are more frequent in UWS than control families (42 and 15%, for UWS and control, respectively). In sum, these data show that affected families were less satisfied with their life than control families (*p* = .025)

Figure 1 shows the distribution of stanine values of FLZ total scores in families with a UWS child and in control families. The corresponding means and standard errors (SE, in parentheses) are 4.23 (0.310) and 3.15 (0.349) for control families and families with a UWS child, respectively. Because the distribution of the values was nearly normal and the variances in the two groups were equal (*p* = 0.22, Levene test), a t-test was applied and confirmed significantly lower life satisfaction in the families with than without a UWS child (t = 2.30, *p* = .025).

### Life situation

For theoretical reasons, answers to FFCv questions were converted into a binary (dichotomous) form: positive (supportive) versus negative (deficient, characterized by a lack of support). More details about dichotomization are given above, Subsection “Questionnaires”. Table 2 shows the distribution of the potentially important factors after their dichotomization (positive versus deficient).

**Table 2:** Number of responses indicating supposedly positive versus negative effect

Variables	Positive cases (%)	Negative cases (%)
Age at the event	13 (50)	13 (50)
Emergency situations	11 (42.3)	15 (57.7)
Help during daytime	15 (57.7)	11 (42.3)
Help during the night^a^	20 (76.9)	6 (23.1)
Family doctor^a^	20 (76.9)	6 (23.1)
Nursing service	10 (38.5)	16 (61.5)
Center of social pediatrics	18 (69.2)	8 (30.8)
Support by health insurance	8 (30.8)	18 (69.2)
Support by nursing care insurance	12 (46.2)	14 (53.8)
Home visits	9 (34.6)	17 (65.4)

Factors marked with ^a^ were not included in the calculation of the total index because of strongly one-sided distributions

**Fig. 2:**
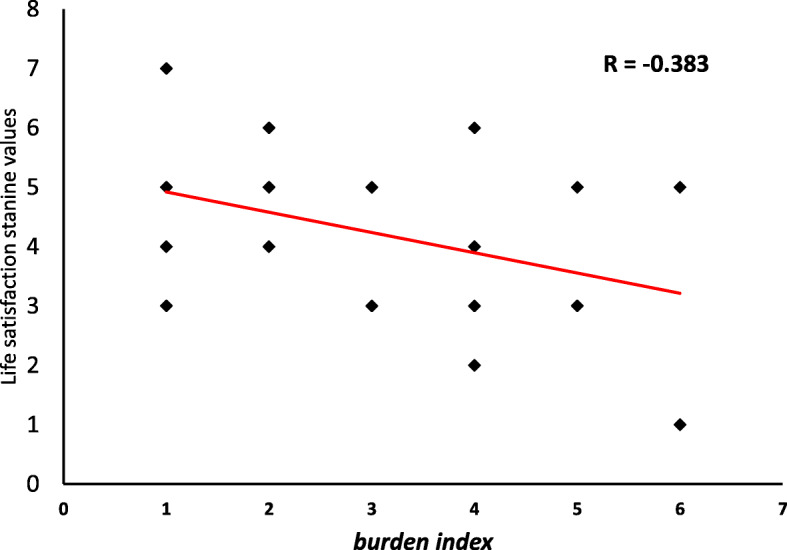
Correlation between the total burden (as estimated on the basis of the anamnestic situation) in families with UWS children, and general life satisfaction (FLZ-SUM). Note that R in the graphic is calculated on the assumption of normal distribution, while the correlation coefficient in the text is distribution-free

The total index of burden for a family with a UWS child was calculated as a sum of the negative (deficient) variables listed in Table 2. The index was negatively related to life satisfaction (Spearman’s rho = −.47, *p* = .015, see Fig. 2).

### Self-management skills in UWS families

**Fig. 3:**
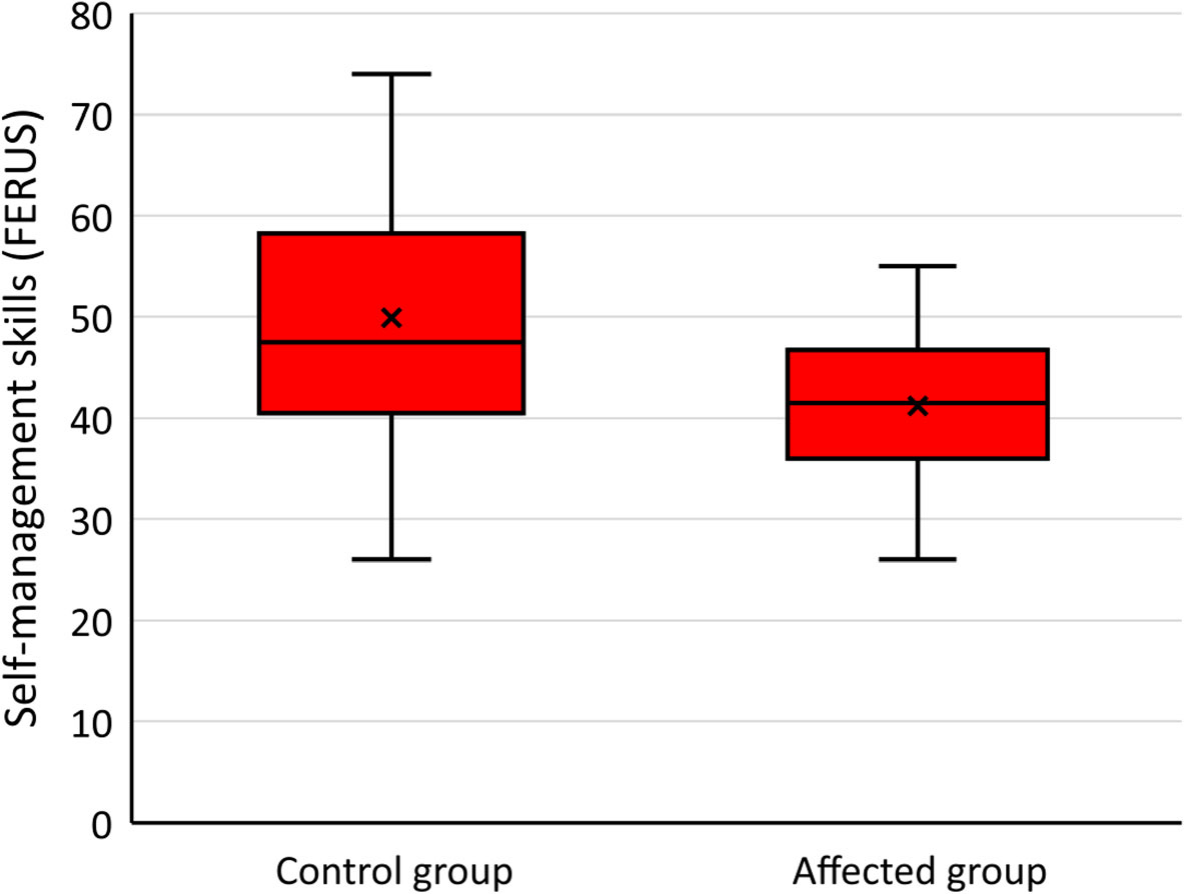
Self-management skills in the families with a UWS child (affected group) and control families

A boxplot diagram illustrating the difference in self-management skills between parents of a UWS affected child and parents of a healthy child is presented in Fig. 3. The t-test indicates that the difference is statistically significant (t = 2.94, df = 40.95 taking in account unequal variances, *p* = .005). Although the doubts cannot be completely ruled out whether all prerequisites for a t-test are fulfilled, the result is further supported by a distribution-free Mann-Whitney test (Z = − 2.54, *p* = .011).

**Fig. 4:**
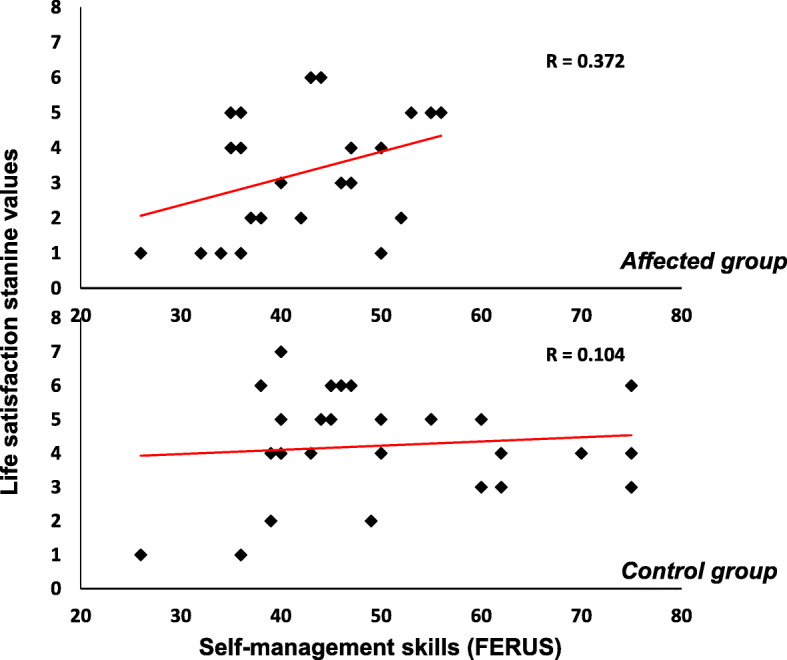
Correlations between self-management skills and the total score of Life Satisfaction in families with a UWS child (bottom panel) and controls families (top panel)

As shown in Fig. 4, self-management skills significantly correlated with overall life satisfaction in the families of UWS children (rho = .531, p = .005). In the control group, on the other hand, the correlation was not significantly different from zero (rho = .078, *p* = .705).

### Life satisfaction and self-acceptance

Our Hypothesis 5 stated that parents of UWS children who are able to accept their emotions are happier than parents who are not. However, the correlation between the overall life satisfaction and the acceptance score for one’s own feelings in the families with UWS children was not significant (Rho = .21; *p* = .3).

### Life satisfaction and emotional regulation

The overall life satisfaction in the affected group was inversely related to experiencing emotional overload (Rho = − .45, *p* = .021). This might be regarded as indirect evidence that the abilities of the families to reduce or compensate for emotional overload yield greater satisfaction with life. However, a more direct test indicated that the correlation between emotion regulation and life satisfaction was not significant (Rho = − 33, *p* = .096).

Furthermore, emotional overload negatively correlated with self-management skills (Rho = −.47, *p* = .020) and emotion regulation (Rho = −.30. *p* = .032). The latter correlation was weak and non-significant in the control group (Rho = −.23; *p* = .26), but substantially higher in the affected group (Rho = −.41, *p* = .036).

While the above findings show several significant correlations with life satisfaction in the group with a UWS child, we do not know how independent the independent variables were. To explore this question, a univariate analysis of variance (ANOVA) was calculated for the affected group. The analysis included the factors Self-Management, Emotions, and Stress, with each factor having (after dichotimization) two levels. The stress situation was taken as the index from Item 26 of the family day questionnaire, and the factor Emotions included acceptance of one’s own emotions, experiencing emotional overload and emotion regulation. The strongest effect on the overall life satisfaction of the families affected by the UWS of their child was the effect of Stress (F (3,23) = 11.02, partial eta-squared η^2^ = .334), followed by Self-management (F (3,23) = 4.44, partial eta-squared η^2^ = .167). Emotional load has little influence on life satisfaction in this model. Because emotional load was negatively and significantly related to self-management (Rho = − 0.47, s.a.), confounding of these two variables can be assumed. Exact ANOVA results are presented in the Supplementary Materials.

### Relationship with a child

The scale KIN “relationship with one’s own children” of the FLZ questionnaire [15] allows us to compare the quality of relationship with children in families with and without a UWS child. The means and standard deviations (in parentheses) were 5.21 (1.91) and 5.50 (1.63), for the affected group and the control group, respectively (t = 0.582, df = 49, *p* = .56), indicating the lack of significant differences.

## Discussion

Life satisfaction is a highly important component of happiness [13]. The issue of life satisfaction (LS) is particularly significant for families who have to cope with special stress such as that related to care of an extremely handicapped child. As Unresponsive Wakefulness Syndrome (UWS) is the most severe chronic neurological disorder altogether [38, 39], a theory-practice transfer is crucial to sustainably and adequately support the families having a child with UWS. The present study was aimed to define some conditions for a happy life of families living together in the home environment with their UWS child.

In accordance with our hypotheses, families with a UWS child rated their overall LS much lower than unaffected families. As expected, their LS was inversely related to the burden of the family (estimated by the number of negative factors) and to emotional overload, and directly related to self-management skills. On the other hand, the hypothesis that the ability to accept one’s emotions should increase LS was not supported by the data. We also expected that the relation to the child would be different in families with a UWS child and in control families, but the analysis of the corresponding scale FLZ-KIN [15] did not reveal any differences.^1^

The data of the ANOVA indicate that the contributions of different factors are of different size. Specifically, all investigated factors together explained about 50% of the variance of general LS, but stress situation alone explained more than 30%. Positive anamnestic situation (in the sense of everyday help, home visits, support by a doctor, nursing service, health insurance, etc.) could be demonstrated as an effect on LS. All these factors are related to a general notion of social support, thus being congruent with numerous data demonstrating the importance of social support for the quality of life of caregivers in various medical conditions (e.g., [46, 49, 64, 70]).

These findings suggest that the simplest way to make families with a UWS child happier is the improvement of their external condition, i.e. providing them more adequate help. Already 70 years ago, Hill [24] proposed a simple formula ABC-X (later developed into a Double ABCX Model: [47]), where A is the stressful situation, B denotes resources available for the family, C is related to family appraisal, and X is the crisis resulting from the three above factors. In these terms, one can state that our data indicate B being more important than C as a factor yielding X.

The result was not completely expected. Germany and Austria, where our respondents came from, belong to a few countries with best-organized social support for severely disabled patients and their families over the world. This concerns the number of hospital beds and doctors (including highly specialized neurologists) per capita, possibilities of insurances and other important factors. Based on these facts we expected that internal factors such as the ability to emotion regulation, or the relation to the ill child, may play at least as important part as the external support (i.e., Hypotheses 3, 5, and 6). This was not the case.

Independently of the powerful factor of external support, self-management skills play a significant part in the determination of LS. Surprisingly, these skills were lower in the families with a UWS child in comparison to the control group. It is possible that the self-management abilities in the former families are exhausted due to excessive stress, as indicated, for example, by the data Ray and Ritchie [53]. This hypothesis finds support in the finding that the negative correlation between self-management skills and LS was observed only in families with a UWS child, but not in control families.

Rolland [57] emphasizes the role of self-management in his Model of Family Adaptation to Chronic Diseases and Disability and relates it to Rotter’s [58] construct of internal control. The conviction that one is able to perform a certain behavior [33] is an essential component of the cognitive-behavioral self-management, and interventions aimed at improvement of the corresponding skills play a substantial role in dumping family stress [9]. Also Sarimski et al. [61] found that perceived parental competence correlated with general self-efficacy and satisfaction with professional support. The satisfaction of the studied families depended on the amount and quality of support, like in the families living with their UWS child in the present study.

While the families with a UWS child had less self-management abilities than the families with healthy children, the self-management skills in the former families strongly affected their LS. This kind of relation between self-management and LS was also shown by Steverink and Lindenberg [65]. They, additionally, indicated that when self-management skills are available, less social resources are needed to attain a high level of life satisfaction. The intriguing question of whether higher self-management skills can compensate for deficient environmental conditions regarding the overall LS could not be answered in our study.

Another component of self-management construct, according to Jack [26], is hope. The Hope scale describes how the individual perceived his/her future. According to Kanfer et al. [28], a growing sense of hope and confidence in the future is important to develop life satisfaction. Particularly, Lukasczik et al. [45] demonstrated the meaning of hope for the well-being of families under chronical stress. In the population of mothers of children with severe physical and mental disabilities, LS has been found to negatively correlate with uncertainty about future [37].

Following Scherer [62] and Gross and Thompson [19], we expected that emotion regulation would correlate with LS. The correlation, however, did not attain the significance level. ANOVA results showed that the impact of emotional factors decreases when external factors (realized through stress) and self-management are included into the model. The lack of correlation between LS and the acceptance of one’s own feelings indicates that the relationship can be more complex. The sooner emotions can be rejected, dismantled, or compensated, the greater the satisfaction in UWS families. The meaning of emotional flooding should be investigated in further studies.

Satisfaction with the relationship to the own child did not differ between the groups (means 5.21 ± 0.39 versus 5.5 ± 0.32 for UWS families and control families, respectively). We believe that this is a positive fact indicating that the parents still regard their child as a full family member notwithstanding the child’s most severe disturbance of consciousness.

In the literature there are only very few comparable studies investigating specific characteristics of families of UWS children. The most relevant study has been published recently by Kluger et al. [32] who examined families of 55 children survived near-drowning. All children had been in UWS at least four weeks after the accident. At the moment of the investigation (between 6.6 and 23.8 years after the accident) all children remained severely disabled and, according to the description, at least six of them were still in UWS. The authors found that health-related quality of life (HRQoL) of the parents did not significantly differ from normal population. The dissimilarity with our findings is, however, difficult to analyze because of the differences in both composition of the target groups and the measurement constructs of HRQoL and LS. On the other hand, negative emotions (particularly guilt) were strongly presented in the families of (formerly) UWS children and negatively correlated with their HEQoL; moreover, 31% of the respondents agreed with the statement that it would have been better if the child died in the acute phase. Kluger et al.’s [32] data further support our conclusion that UWS children are still regarded as valuable family members.

Also Giovanetti et al. [18] reported high level of daily stress in a group of 35 parents (mostly mothers) of children with very severe disorders of consciousness (UWS or Minimally Conscious State [MCS]). The levels of depression, state and trait anxiety of respondents were heightened as compared with the normative data. It should be mentioned that the study did not include a control group, and the data of the parents of ill children were directly compared with population statistics; it is further unclear what portion of the children lived together with their families, which was one of the main inclusion criteria in the current study. A curious difference is that Giovanetti et al.’s sample included thirty-three (i.e., as many as 94%) respondents with the educational status of high school or above, as compared with 14 (58%) of such respondents in our sample (Yates corrected chi-square = 11.60, df = 2, *p* = .0007). One may therefore suppose that the Italian sample was biased toward highly-educated participants.

Although Doege et al. [14] did not study UWS children, their results are of relevance as they investigated a large sample of 327 families of children with severe intellectual disabilities. They described a very high burden of caregivers on the border of exhaustion, and revealed family coherence and high self-esteem as the main stress-reducing factors. These factors were unrelated to the depth of the child’s disabilities. Likewise, Giovanetti et al. [18] did not find a significant impact of the child’s diagnosis on the emotional state of parents of UWS and MCS children. These data indicate that the severity of the child’s condition may, within a certain range, be not a decisive factor determining the burden of the family (e.g., [30, 66]; although some features of stress can depend on children’s diagnosis: [23]). In summary, both Doege et al. [14] and the present study show that families affected by a severe mental disorder of their child would benefit from a combination of positive external conditions (in the present case, social and logistic support) and personality traits and styles (e.g., self-management skills). A similar conclusion can be drawn from the results of Gschwendt et al. [21], who studied stress conditions of teenage mothers (14–20 years of age) and their toddlers (12–17 months of age).

Being one of the very first studies devoted to the problems of families of UWS children, the present study is, of course, not free from serious limitations. First, the response rate of about 16% was rather low. The average response rate for mail surveys has been reported as high as 50% [42]. However, this number is an average over many surveys, most of which with only 10 to 50 items, and the response rate is known to strongly and negatively correlate with the number of items. Furthermore, the response rate is usually low in highly stressed populations (e.g., high-level managers), and the population of parents with UWS children also belongs to this category. Low response rates are dangerous because they may sometimes indicate a bias, when individuals agreeing and rejecting to respond differ in some important feature(s). One might suppose, for example, that parents receiving sufficient support would have more time and be ready to respond, while those without support would be overburdened and reject participation. Our data indicate that this bias is rather improbable because several respondents reported to have no support and being overburdened, but yet they answered. This fact cannot, however, rule out possibilities of other biases.

To increase the response rate in further studies, we would suggest, firstly, to use shorter questionnaires, and secondly, to organize the interviews in form of personal contacts. The latter option, theoretically the best one, requires considerable resources because the investigators personally visit each family, up to two hundred in the German speaking space.

Like in many similar studies, we were limited by our own hypotheses, and these, in turn, were limited by the reasons based on previous (similar) studies. Different hypotheses would result in application of different methodological tools, which, perhaps, would open a better understanding of the problems of UWS families.

Our study was limited in covering some potentially important aspects of family life. Only few participants were fathers. The bias toward female respondents is, unfortunately, common in similar studies (e.g., [18]). We asked only a few questions about the condition of patients’ siblings, and we did not obtain any response from siblings of UWS patients, although many of them would have been able to respond. A descriptive study of three families with MCS patients indicate that siblings may be strongly involved in family situation [44]. Future studies should take these aspects into account and, possibly, explicitly ask patients’ siblings to report their view.

Finally, we should keep in mind that all reported data were subjective. If a participant reported, e.g., that the child had been hospitalized every year, we just took this datum and did not check it. We cannot rule out that a different result might be obtained if the situation of UWS patients is depicted by a third-party observer.

It should be noted that overcoming these limitations is very challenging and probably cannot be done in a single study. For example, if we want to increase response rate, we should limit the expected time of answering, thus restricting the volume of information. On the other hand, if we are interested in more various aspects of family life, and want to test more hypotheses, we should ask more questions, which may result in a decrease of the response rate. Thus, different challenges stay in a trade-off relation to each other, and finding a right balance is not an easy job.

## Conclusion

The present results show that happy life is possible in a family living together with a child who has a UWS. The most important condition for this is a sufficient social and logistic support that curbs the multiple stress experienced by such families. This conclusion is less trivial that it may seem, given that the data were collected in Germany and Austria, where the level of support is already much better than in many other countries of the world. Therefore, we may expect that the impact of this factor could be even stronger in other populations. As regards psychological factors, our results emphasize the importance of self-management skills in the families with a UWS child. Psychological help should be aimed at improvement of self-management abilities as well as the development of strategies to avoid emotional overflow.

## Supplementary Information

**Additional file 1.** English Translation of German-language Questionnaires.

**Additional file 2.** Supplement 2. Frequency tables.

**Additional file 3.** Supplement 3. Table III. Results of the ANOVA for overall Life Satisfaction.

## Data Availability

The data can be obtained from Dr. Christiane Chadasch (e-mail see title page) on reasonable request.

## Abbreviations

FERUS: Resource and self-management capability questionnaire (Jack, 2007)
FFCv: Questionnaire on everyday family life with a UWS child (Chadasch, 2011)
FLZ: Questionnaire on life satisfaction (Fahrenberg et al., 2000)
HRQoL: Health-related quality of life
LS: (generalized) life satisfaction
MCS: Minimally conscious state
SE: Standard error
SEE: Scales for experiencing emotions (Behr and Becker, 2004)
UWS: Unresponsive wakefulness syndrome

## Footnotes

1 One might suppose that the relationship to a UWS child can be changed in different directions in different affected families, which would result in the apparent zero change on average. However, in this case the data of affected families would have a larger variance as compared with control families, and this was also not the case.

## References

[1] Antonovsky A (1979). Health, stress and coping.

[2] Antonovsky A (1987). Unraveling the mystery of health: how people manage stress and stay well.

[3] Ashwal S, Bale JF, Coulter DL, Eiben R, Garg BP, Hill A, Myer EC, Nordgren RE, Shewmon A, Sunder TR, Walker RW (1992). The persistent vegetative state in children: report of the child neurology society ethics committee. Ann Neurol.

[4] Baldwin S (2016). The costs of caring. Families with disabled children.

[5] Bandura A (1977). Self-efficacy: toward a unifying theory of behavioral change. Psychol Rev.

[6] Barlow J, Swaby L, Turner A (2008). Perspectives of parents and tutors on a self-management program for parents/guardians of children with long-term and life-limiting conditions: “A life raft we can sail along with”. J Community Psychol.

[7] Beavers J, Combrinck-Graham L (1989). Physical and cognitive handicaps. Children in family contexts. Perspectives on treatment.

[8] Behr, M., Becker, M. SEE*.* Skalen zum Erleben von Emotionen. Manual. Göttingen: Hogrefe; 2004*.*

[9] Boss P (2002). Family stress management.

[10] Braakman R, Jennett WB, Minderhoud JM. Prognosis of the posttraumatic vegetative state. Acta Neurochirurgica. 1988;95:49–52 10.1007/BF01793082 3218553

[11] Chadasch, C. FFCv*.* In: Chadasch, C. (2016). Zusammenleben und Lebenszufriedenheit in Familien mit einem kind im Zustand des coma vigile. Eine empirische Studie über Familien am Rande der Kraft und ihre Ressourcen. https://kups.ub.uni-koeln.de/6565/ the datasets used and analysed during the current study are available from the corresponding author on request 2011.

[12] de Ridder D, Schreurs K. Developing interventions for chronically ill patients: is coping a helpful concept? Clin Psychol Rev. 2001;21:205-40. 10.1016/s0272-7358(99)00046-x. 10.1016/s0272-7358(99)00046-x 11293366

[13] DeNeve KM, Cooper H (1998). The happy personality: a meta-analysis of 137 personality traits and subjective well-being. Psychol Bull.

[14] Doege D, Aschenbrenner RM, Nassal A, Holtz K-L, Retzlaff R (2011). Familienkohärenz und Resilienz bei Eltern von Kindern mit intellektueller Behinderung. Zeitschrift für Gesundheitspsychologie.

[15] Fahrenberg, J., Myrtek, M., Schumacher, J., Brähler, E. Fragebogen zur Lebenszufriedenheit*. FLZ. Handanweisung.* Göttingen: Hogrefe; 2000.

[16] Faul F, Erdfelder E, Lang A, Buchner A (2007). G*power 3: A flexible statistical power analysis program for the social, behavioral and biomedical science. Behav Res Methods.

[17] Geremek A (2009). Wachkoma. Medizinische, rechtliche und ethische Aspekte.

[18] Giovannetti AM, Pagani M, Sattin D, Raggi A, Strazzer S, Castelli E, Trabacca A, Martinuzzi A, Leonardi M l. Children in vegetative state and minimally conscious state: patients' condition and caregivers' burden. Sci World J. 2012;232149. 10.1100/2012/232149. 10.1100/2012/232149 PMC3290454 22454603

[19] Gross JJ, Thompson RA, Gross JJ (2007). Emotion regulation: conceptual foundations. Handbook of emotion regulation.

[20] Grootenhuis M, Last BF (1997). Adjustment and coping by parents of children with cancer: A review of the literature. Support Care Cancer.

[21] Gschwendt, M. A. Early manifestations of aggression in infants of high risk mother-infant dyads. PhD Thesis, University of Potsdam 2002. https://publishup.uni-potsdam.de/opus4-ubp/frontdoor/deliver/index/docId/48/file/gschwend.pdf, called on Jan 16, 2020.

[22] Heindl UT, Laub MC (1996). Outcome of persistent vegetative state following hypoxic or traumatic brain injury in children and adolescents. Neuropediatrics.

[23] Hentinen M, Kungäs H (1998). Factors associated with the adaptation of parents with a chronically ill child. J Clin Nurs.

[24] Hill R (1949). Families under Stress.

[25] Howard, J. An intimate view of the ‘super parents’ of chronically ill children. *CNN*. 2016 http://www.cnn.com/2016/12/07/health/chronically-ill-children-super-parents/, called on Jan 16, 2020.

[26] Jack, M. FERUS*. Fragebogen zur Erfassung von Ressourcen und Selbstmanagementfähigkeiten.* Göttingen: Hogrefe; 2007.

[27] Kampfl A, Schmutzhard E, Franz G, Pflauser B, Haring H-P, Ulmer H, Felber S, Golaszewski S, Aichner F (1998). Prediction of recovery from post-traumatic vegetative state with cerebral magnetic-resonance imaging. Lancet.

[28] Kanfer FH, Gaelick-Buys L, Kanfer FH, Goldstein AP (1991). Self-management methods. Helping People Change: A Textbook of Methods (Pergamon General Psychology Series, Vol. 52).

[29] Kant, I. Groundwork of the Metaphysics of Morals. Trans. M. J. Gregor. Cambridge: Cambridge University press (1st ed. 1785) 2011.

[30] Kazak A (1987). Families with disabled children: stress and social networks in three samples. J Abnorm Child Psychol.

[31] Kemp V (2011). Use of ‘chronic disease self-management strategies’ in mental healthcare. Curr Opin Psychiatry.

[32] Kluger GJ, Kirsch A, Hessenauer M, Aust H, Berweck S, Sperl W, Betzler C, von Stülpnagel-Steinbeis C, Staudt M (2019). Unresponsive wakefulness syndrome in children after near-drowning: long-term outcome and impact on the families. Neuropediatrics.

[33] Koenig, C. J., Kleinmann, M. (2006). Selbstmanagement. In: Schuler, H. (Hsgb.). *Lehrbuch der Personalpsychologie*, 2nd Ed., Göttingen. 329-348.

[34] Kotchoubey B, Squire L (2009). Vegetative state. Encyclopedia of neuroscience.

[35] Kotchoubey B, Lang S, Mezger G, Schmalohr D, Schneck M, Semmler A, Bostanov V, Birbaumer N (2005). Information processing in severe disorders of consciousness: vegetative state and minimally conscious state. Clin Neurophysiol.

[36] Kotchoubey B, Pavlov YG (2018). A systematic review and meta-analysis of the relationship between brain data and the outcome in disorders of consciousness. Front Neurol.

[37] Küçük EE, Alemdar DK (2018). Life satisfaction and psychological status of mothers with disabled children: A descriptive study. Community Ment Health J.

[38] Laureys S, Owen AM, Schiff ND (2004). Brain function in coma, vegetative state and related disorders. Lancet Neurol.

[39] Laureys, S., Celesia, G. G., Cohadon, F., Lavrijsen, J., León-Carrión, J., Sannita, W. G., Sazbon, L., Schmutzhard, E., von WIld, K R., Zeman, A., Dolce, G. (2010). Unresponsive wakefulness syndrome: A new name for the vegetative state or apallic syndrome? BMC Med*,* 8(68). 10.1186/1741-7015-8-68 PMC2987895 21040571

[40] Lavrijsen JC, van den Bosch JS, Koopmans RT, van Weel C (2005). Prevalence and characteristics of patients in a vegetative state in Dutch nursing homes. J Neurol Neurosurg Psychiatry.

[41] Li-Tsang CWP, Yau MK, Yuen HK (2001). Success in parenting children with developmental disabilities: some characteristics, attitudes and adaptive coping skills. Brit J Developmental Disabilities.

[42] Lindemann, N. (2019). What’s the average survey response rate? [2019 benchmark] https://surveyanyplace.com/average-survey-response-rate/, called on 13/12/2020.

[43] Lorig KR, Holman HR (2003). Self-management education: history, definition, outcomes, and mechanisms. Ann Behav Med.

[44] Løvstad, M., Solbraekke, K. N., Kirkevold, M., Geard, A., Hauger, S. L., Schanke, A.-K. (2018). “It gets better. It can ´t be worse than what we have been through.” Family accounts of the minimally conscious state. Brain Injury, doi: 10.1080/02699052.2018.1539244, www.tandfonline.com/loi/ibij20. 10.1080/02699052.2018.1539244 30351974

[45] Lukasczik M, Gerlich C, Musekamp G, Saupe-Heide M, Löbmann R, Vogel H, Neuderth S (2014). Externe Qualitätssicherung in Einrichtungen der stationären Vorsorge und Rehabilitation für Mütter und Väter einschließlich Mutter−/Vater-Kind-Einrichtungen: Einrichtungsvergleichende Analysen im Bereich Ergebnisqualität. Gesundheitswesen.

[46] Matuz T, Birbaumer N, Hautzinger M, Kübler A (2010). Coping with amyotrophic lateral sclerosis: an integrative view. J Neurol Neurosurg Psychiatry.

[47] McCubbin HI, Patterson JM, McCubbin HI, Gaubic AE, Patterson JM (1982). The family stress process: the double ABCX model of adjustment and adaptation. Family stress, coping, and social support.

[48] Melnyk BM, Feinstein NF, Moldenhouer Z, Small L (2001). Coping in parents of children who are chronically ill: strategies for assessment and intervention. Pediatr Nurs.

[49] O’Boyle CA, Waldron D (1997). Quality of life issues in palliative medicine. J Neurol.

[50] Patterson JM (2002). Understanding family resilience. J Clin Psychol.

[51] Rabow, M. W., Hauser, J. M., Adams, J. Supporting family caregivers at the end of life: »they don't know what they don't know«. JAMA*.* 2004 291; 483–491. 10.1001/jama.291.4.483 14747506

[52] Rafferty KA, Hutton K, Heller S (2019). “I will communicate with you, but let me be in control”: understanding how parents manage private information about their chronically ill children. Health Commun.

[53] Ray LD, Ritchie JA (1993). Caring for chronically ill children at home: factors that influence parents' coping. J Pediatr Nurs.

[54] Reichman NE, Corman H, Noonan K (2008). Impact of child disability on the family. Matern Child Health J.

[55] Retzlaff R (2010). Familien-Stärken. Behinderung, Resilienz und systemische Therapie.

[56] Rogers C. A theory of therapy, personality and interpersonal relationships as developed in the client-centered framework. In: Koch S (Ed.). Psychology: A Study of a Science, Vol. 3. New York: McGraw Hill; 1959.

[57] Rolland JS, Gehlert S, Browne T (2019). Families, health, and illness. Handbook of health social work.

[58] Rotter, J. B. Generalized expectancies for internal versus external control of reinforcement. Psychological Monographs. 1966; 80. 5340840

[59] Ryan P, Sawin KJ (2009). The individual and family self-management theory: background and perspectives on context, process, and outcomes. Nurs Outlook.

[60] Salovey P, Mayer JD (1990). Emotional intelligence. Imagin Cogn Pers.

[61] Sarimski, K. Hintermair, M., Lang, M. Familienorientierte Frühförderung von Kindern mit Behinderung. München – Basel: Ernst Reinhardt Verlag 2013.

[62] Scherer KR (1982). Emotion as a process: function, origin and regulation. Soc Sci Inf.

[63] Scorgie K, Wilgosh L, McDonald L (1998). Stress and coping in families of children with disabilities: an examination of recent literature. Developmental Disabilities Bull.

[64] Sommer G, Fydrich T (1989). Soziale Unterstützung.

[65] Steverink N, Lindenberg S (2008). Do good self-managers have less physical and social resource deficits and more well-being in later life?. Eur J Ageing.

[66] Thompson RJ, Gustafson KE, Hamett KW, Spork A (1992). Stress, coping, and family functioning. J Pediatr Psychol.

[67] van Erp WS, Lavrijsen JCM, van de Laar FA, Vos PE, Laureys S, Koopmansa RTCM. The vegetative state/unresponsive wakefulness syndrome: A systematic review of prevalence studies. Eur J Neurol. 2014. 10.1111/ene.12483. 10.1111/ene.12483 25039901

[68] van Erp WS, Lavrijsen JCM, Vos PE, Bor H, Laureys S, Koopmansa RTCM (2015). The vegetative state: prevalence, misdiagnosis, and treatment limitations. J Am Med Directors Assoc.

[69] van Erp WS, Lavrijsen JCM, Vos PE, Laureys S, Koopmansa RTCM (2020). Unresponsive wakefulness syndrome: outcomes from a vicious circle. Neurology Grand Rounds.

[70] Willits FK, Crider DM (1988). Health rating and life satisfaction in the later middle years. J Gerontol.

[71] Yau KM, Li-Tsang CWP (1999). Adjustment and adaptation in parents of children with developmental disability in two-parent families: A review of the characteristics and attributes. Brit J Developmental Disabilities.

