# Supplementary material for: Life satisfaction in families with a child in an Unresponsive Wakefulness Syndrome
Source: BMC Pediatr. 2021 Mar 8;21:116. doi: 10.1186/s12887-021-02549-8 (PMC7938537; doi:10.1186/s12887-021-02549-8)
Supplement: Supplementary file 1 — Additional file 1. English Translation of German-language Questionnaires. [file 12887_2021_2549_MOESM1_ESM.docx]

**Supplementary Materials**

**English Translation of German-language Questionnaires**

NOTE! The translations are not adjusted for English speaking population. Their only aim is to give a non-German speaking reader an idea of what was asked. They must not be used in any study.

**Fragebogen zur Erfassung von Ressourcen und Selbstmanagementfähigkeiten (FERUS)** (Jack, 2007. Göttingen: Hogrefe)

**Questionnaire for assessment of resources and self-management skills**

*Quality information:* All scales have a test-retest reliability between .66 and .86, an internal consistency between .86 and .93, and high content validity (Jack, 2007). T-values, mean values, standard deviations, confidence intervals, and critical differences are available in Scherm (2002).

**Name: (Never asked)**

**Age:**

**Gender:** o male o female

**Date:**

Education (if your degree is not listed here, please tick the most comparable):

o no education

o special school education

o secondary school leaving certificate

o high school diploma

o (Technical) university entrance qualification

**Instruction**

A number of statements are given below. These statements refer to the **period of the last 2-3 weeks, including today,** based to your current situation. Please tick the most relevant answers.

The numbers 1-5 in the answer box mean

| (1)  fully disagree | (2)  rather disagree | (3)  neither agree nor disagree | (4)  rather agree | (5)  completely agree |
| --- | --- | --- | --- | --- |

Task example

I have the ability to solve my problems, when I try.

| 1 | 2 | 3 | 4 | 5 |
| --- | --- | --- | --- | --- |

The tick in the answer box means that you fairly agree with the statement and that you rate its problem-solving ability to be quite good.

Below you can read the translation of the questions. In the original, there are five boxes each.

1. The things I am most concerned with at the moment, will be of use to me in the future.
2. If someone stands in the way of realizing my intensions and goals, I will still find a way to promote my ideas.
3. When there is a conflict that I cannot change, I remain calm and deal with the problem.
4. When I fail, I look at my behavior in detail and try to understand what was going wrong.
5. I have to try out at home new behaviors that I learn or have learnt in therapy so that I feel better psychologically and/or physically in the future.
6. If I don´t change something in my life situation, it becomes more and more difficult in my private sphere.
7. In order to achieve a difficult aim, I develop a detailed plan beforehand how I have to act.
8. In conflict situations, I do not give up and at least try to find a compromise.
9. I am able to solve my problems if I try.
10. I look forward to my future with optimism.
11. When I cannot change a situation, I try to make the best of it.
12. When I want to achieve a goal, and get into conflict with someone because of it, I try to get that person to help me.
13. If I ever lose heart, I try to think about my successes to give me courage again.
14. I have friends and / or relatives who also listen carefully to me when I have a problem.
15. I feel able to achieve my intentions and goals.
16. If something goes wrong again and there is nothing I can do about it, I try to adapt to the situation.
17. If I don't change something in my life situation, my job will become more and more difficult.
18. In order to deal with a problem, I first have to get an accurate picture of the whole situation.
19. Even if everything goes wrong, I believe that things will turn better again.
20. I have friends and / or relatives who just give me a hug.
21. To get rid of my bad feelings after failure, I tell myself that it is not a catastrophe and that I need to keep going to be successful.
22. In difficult situations, I usually behave correctly.
23. When I am in a difficult situation, I sometimes seek advice to help me understand what to do about it.
24. I think I'll have enough time in my life to do things that I enjoy.
25. If there are problems that cannot be dealt with immediately, I try to wait calmly.
26. If I want to achieve an important goal, I look at how I can best cope with the situation with my behavior.
27. When I get scared in a situation, I say, "Keep calm, it's not a disaster."
28. If I'm sick, I can without hesitation ask friends or relatives to do certain things for me (e.g. shopping, getting medication).
29. If I don't achieve a goal, I try to find out if I have to change my behavior in order to get there.
30. Even if events or my problems roll over and over, I think I can cope with them.
31. In problematic situations, I often think calmly and draw up a plan to deal with the problem.
32. I would like to change my current private or professional situation because I am dissatisfied with many things.
33. Whenever my problems overwhelm me again, I say to myself: "Cheer up, it's not that bad".
34. I want to learn how to deal better with difficult situations.
35. If I want to talk about a problem, I know whom to go to.
36. Whatever happens, I know how to help myself or get help.
37. Sometimes I leave things and problems as they are and can deal with them calmly.
38. I would like to be able to solve my problems with the things that I learn or have learned in therapy.
39. Even when I feel at the bottom, I try to say encouraging things to myself.
40. In difficult situations, I usually come up with something creative to change the situation for the better.
41. With some friends and / or relatives I can be completely left out without hesitation.
42. I usually have a good solution to my problems.
43. In order to solve a problem, it is important for me to know exactly my own behavior in this situation.
44. Whenever I have severe anxiety or depression, I think to myself: "That's okay, everyone has that”.
45. There are things in my life that I would like to change because it is no longer satisfying for me as it was before.
46. I think the life will still hold many opportunities for me.
47. Even when I am very tense from a problem, I know how to relax and deal with it again.
48. With this therapy, I have taken the first step in the right direction and now I want to change my life in some areas.
49. To solve a problem, I often come up with a plan to resolve it.
50. There are people who really help me when I don't know what to do next.
51. When problems arise, I know I have the ability to deal with them.
52. My current experience is a good preparation for dealing with future problems.
53. It is very important to me to learn new behaviors in order to be able to deal better with problems and conflicts.
54. Even if I make a mistake, there are people who stick with me.
55. When problems arise, all I have to do is to ponder, and I usually have several ideas on how to deal with them.
56. I have great hopes for the future.
57. I think that the therapy (in the clinic) helps or has helped me to learn behaviors that I can also use at home in order to cope better with some areas of life.
58. I have a circle of friends to which I feel I belong.
59. I know that I have to change something about myself in the future so that I can improve mentally and / or physically.
60. I will see many more beautiful things in my life.
61. When I want to get rid of a bad habit, I first try to figure out the reasons that support it.
62. There is a very familiar person whose help I can always rely on.
63. I really want to change some things in my life so that I can find joy in life again.
64. I believe that I can achieve the things that I want.
65. There are people to whom I can show my feelings without being embarrassed.
66. Some things that lie ahead of me are hard to deal with, but I'll do this.

**Skalen zum Erleben von Emotionen (SEE)**

**Scales of emotional experience**

(Behr & Becker, 2004. Göttingen: Hogrefe)

*Quality information:* For a time-economic application, 42 items have been recommended in an optimization study with N = 456 (Behr et al., 2002). The internal consistency of individual scales varies between .70 and .86. The pairwise intercorrelations between the scales (the average is .22, and the highest one, .46) are low enough to regard the scales as mutually independent. Where the scales intercorrelate, this is in line with theory-driven expectations (Behr and Becker, 2004). The convergent and discriminant validity of SEE was tested using its correlations with various instruments such as Five Factor Inventory (Costa and McCrae, 1992) and Frankfurt self-concept scales (Deusinger, 1982).

Date:

Name: (never asked)

Gender: male

female

Age:

Denomination:

Native language:

Education:

Completed before the last secondary school class

Finished with the last secondary school class

Middle or commercial school without a final exam

Middle or commercial school with final exam

High school without high school certificate

High school with certificate but without further education

High school with incomplete college/university

College/university degree

On the following pages, you will find statements about the perception of and dealing with feelings. For each statement, you can tick how much it applies to you personally. There are no right or wrong answers. Everything what applies to you personally is right. Please do not try to make the "best impression", but answer basing on your personal feelings. Even if a question is not exactly tailored to you personally, please tick each question as best as possible.

Please tick one of the five possible answers:

| Not true at all | Rather not true | Somewhat true | Pretty much true | Totally true |
| --- | --- | --- | --- | --- |

##

1. My feelings become clearer to me through my dreams.
2. I always know how I can control my emotional state.
3. I have some feelings that I would better not have.
4. My feelings are often reflected in my body sensations.
5. My self-control leaves a lot to be desired.
6. My fantasies help me to cope with the past.
7. I stand to all my feelings.
8. I notice my body signals rapidly.
9. Thank God I have my feelings under control.
10. Feelings of internal tension, relaxation, etc. help me to make decisions.
11. I wish I weren't always tossed around by my emotions.
12. I frequently ignore my inner experience.
13. It often helps me to indulge in daydreams when I am under stress.
14. If I want to get into a better, more lively mood, I can influence myself.
15. Even when I am seething inside, I can appear calm from the outside.
16. I feel what I feel and that's ok.
17. I often wish I could perceive my feelings better.
18. I find daydreaming useful.
19. My physical condition mostly corresponds to my mental condition.
20. Sometimes I am so full of emotion that I cannot stand it.
21. When I'm excited, I usually know how to calm down.
22. When making decisions, I rely on my bodily sensations.
23. There is no question for me that I have a right to all my feelings.
24. I am always in control of myself.
25. Sometimes I get angry with my feelings.
26. My daydreams give me clues about my needs and desires.
27. Sensations such as palpitations, stomach pressure, skin tingling give me good orientation of what I want.
28. I wish I could turn my mind off sometimes.
29. I would like to experience more inside.
30. I am not ashamed of my feelings.
31. I have a lot of feelings inside that I would like to get rid of.
32. If I feel uncomfortable in a situation, then I notice this e.g. in the stomach, on the skin, in muscle tone or something similar.
33. Unfortunately, when I'm seething inside, my environment notices it immediately.
34. All my feelings are allowed to be exactly as they are.
35. I often perceive physical complaints as an expression of emotional discomfort.
36. I often do not feel the signals from my body.
37. Too often I get overwhelmed by my feelings.
38. My dreams help me to better understand my relationships with others.
39. If I want, I can manipulate my emotions.
40. As a rule, others cannot see what is going on in me.
41. For me, my intuition has a lot to do with the perception of my body.
42. Whatever feelings arise in me, I can always accept first.

**Fragebogen zur Lebenszufriedenheit (FLZ)** (Fahrenberg, Myrtek, Schumacher & Brähler, 2000. Göttingen: Hogrefe)

Life Satisfaction Questionnaire. Primary data from the standardization sample 1994.

*Quality information:* The questionnaire was tested in a population-representative data (N = 2870), checked for test quality criteria and normalized for different age and gender groups. The reliability (Cronbach's Alpha) was between .82 and .94. Content validity and logical validity of the FLZ was examined by Fahrenberg et al. (2000) based on correlations with self-assessment measures, sociodemographic features, and peer group data (Pavot et al., 1991).

Instruction: For each of the statements on the following pages, please tick the number that most closely corresponds to your satisfaction with the statement in question.

Please tick only one box for each statement.

|  | **Health** | **1 very dissatisfied** | **2 dissatisfied** | **3 rather dissatisfied** | **4 neither/nor** | **5 rather satisfied** | **6 satisfied** | **7 very satisfied** |
| --- | --- | --- | --- | --- | --- | --- | --- | --- |
| 1 | With my general physical health, I am ... |  |  |  |  |  |  |  |
| 2 | With my mental state, I am … |  |  |  |  |  |  |  |
| 3 | With my physical condition, I am … |  |  |  |  |  |  |  |
| 4 | With my intellectual capacity, I am … |  |  |  |  |  |  |  |
| 5 | With my resistance to disease, I am … |  |  |  |  |  |  |  |
| 6 | When I think of how often I have pain, then I am … |  |  |  |  |  |  |  |
| 7 | When I think of the number of times I´ve been sick, then I am … |  |  |  |  |  |  |  |

|  | **Job and occupation** (Retired people: please rate previous occupation) | **1 very dissatisfied** | **2 dissatisfied** | **3 rather dissatisfied** | **4 neither/nor** | **5 rather satisfied** | **6 satisfied** | **7 very satisfied** |
| --- | --- | --- | --- | --- | --- | --- | --- | --- |
| 8 | With my position at my job, I am ... |  |  |  |  |  |  |  |
| 9 | When I think about my confidence in my professional future, then I am … |  |  |  |  |  |  |  |
| 10 | With the success that I have in my job, I am … |  |  |  |  |  |  |  |
| 11 | With the career opportunities that I have in my workplace, I am … |  |  |  |  |  |  |  |
| 12 | With the working atmosphere at my workplace, I am … |  |  |  |  |  |  |  |
| 13 | As far as the extent of my occupational demands and stresses is concerned, I am … |  |  |  |  |  |  |  |
| 14 | With the variety that my job offers, I am … |  |  |  |  |  |  |  |

|  | **Financial situation** | **1 very dissatisfied** | **2 dissatisfied** | **3 rather dissatisfied** | **4 neither/nor** | **5 rather satisfied** | **6 satisfied** | **7 very satisfied** |
| --- | --- | --- | --- | --- | --- | --- | --- | --- |
| 15 | With my income / wages, I am ... |  |  |  |  |  |  |  |
| 16 | With what I own today, I am … |  |  |  |  |  |  |  |
| 17 | With my standard of living, I am … |  |  |  |  |  |  |  |
| 18 | With the security of my economic existence, I am … |  |  |  |  |  |  |  |
| 19 | With my future earning potential, I am … |  |  |  |  |  |  |  |
| 20 | With the opportunities that I can offer my family due to my financial situation, I am … |  |  |  |  |  |  |  |
| 21 | With my expected (financial) old-age insurance, I am … |  |  |  |  |  |  |  |

|  | **Leisure** | **1 very dissatisfied** | **2 dissatisfied** | **3 rather dissatisfied** | **4 neither/nor** | **5 rather satisfied** | **6 satisfied** | **7 very satisfied** |
| --- | --- | --- | --- | --- | --- | --- | --- | --- |
| 22 | With the length of my annual vacation, I am ... |  |  |  |  |  |  |  |
| 23 | With the length of my evenings and weekends, I am … |  |  |  |  |  |  |  |
| 24 | With the relaxation value of my annual vacation, I am ... |  |  |  |  |  |  |  |
| 25 | With the relaxation value of my evenings and weekends, I am … |  |  |  |  |  |  |  |
| 26 | With the amount of time I have for my hobbies, I am … |  |  |  |  |  |  |  |
| 27 | With the time I can devote to people close to me, I am … |  |  |  |  |  |  |  |
| 28 | With the variety in my free time, I am … |  |  |  |  |  |  |  |

|  | **Marriage and partnership**  *(Please only fill in if you have a permanent partner)* | **1 very dissatisfied** | **2 dissatisfied** | **3 rather dissatisfied** | **4 neither/nor** | **5 rather satisfied** | **6 satisfied** | **7 very satisfied** |
| --- | --- | --- | --- | --- | --- | --- | --- | --- |
| 29 | With the demands that my marriage / partnership place on me, I am ... |  |  |  |  |  |  |  |
| 30 | With our joint ventures, I am … |  |  |  |  |  |  |  |
| 31 | With the honesty and openness of my (spouse) partner, I am ... |  |  |  |  |  |  |  |
| 32 | With the understanding that my (spouse) partner shows, I am … |  |  |  |  |  |  |  |
| 33 | With the tenderness and affection that my (spouse) partner shows me, I am … |  |  |  |  |  |  |  |
| 34 | With the security that my (spouse) partner gives me, I am … |  |  |  |  |  |  |  |
| 35 | With the willingness to help me that my (spouse) partner shows me, I am … |  |  |  |  |  |  |  |

|  | **Relationship to one´s own children**  *(Only fill in if you have children of your own)* | **1 very dissatisfied** | **2 dissatisfied** | **3 rather dissatisfied** | **4 neither/nor** | **5 rather satisfied** | **6 satisfied** | **7 very satisfied** |
| --- | --- | --- | --- | --- | --- | --- | --- | --- |
| 36 | When I think about how my kids and I get along, I am ... |  |  |  |  |  |  |  |
| 37 | When I think of my children´s educational and professional advancement, I am … |  |  |  |  |  |  |  |
| 38 | When I think about how much fun I have with my children, I am ... |  |  |  |  |  |  |  |
| 39 | When I think about the troubles and costs my children have cost me, I am … |  |  |  |  |  |  |  |
| 40 | With the influence that I have on my children, I am … |  |  |  |  |  |  |  |
| 41 | With the recognition that my children show me, I am … |  |  |  |  |  |  |  |
| 42 | With our joint ventures, I am … |  |  |  |  |  |  |  |

|  | **Own person** | **1 very dissatisfied** | **2 dissatisfied** | **3 rather dissatisfied** | **4 neither/nor** | **5 rather satisfied** | **6 satisfied** | **7 very satisfied** |
| --- | --- | --- | --- | --- | --- | --- | --- | --- |
| 43 | With my abilities and skills, I am ... |  |  |  |  |  |  |  |
| 44 | With the way I´ve lived my life so far, I am … |  |  |  |  |  |  |  |
| 45 | With my outward appearance, I am ... |  |  |  |  |  |  |  |
| 46 | With my confidence and self-assurance, I am … |  |  |  |  |  |  |  |
| 47 | With my character / being, I am … |  |  |  |  |  |  |  |
| 48 | With my vitality (zest for life), I am … |  |  |  |  |  |  |  |
| 49 | When I think about how I get along with other people, I am … |  |  |  |  |  |  |  |

|  | **Sexuality** | **1 very dissatisfied** | **2 dissatisfied** | **3 rather dissatisfied** | **4 neither/nor** | **5 rather satisfied** | **6 satisfied** | **7 very satisfied** |
| --- | --- | --- | --- | --- | --- | --- | --- | --- |
| 50 | With my physical attractiveness, I am ... |  |  |  |  |  |  |  |
| 51 | With my sexual performance, I am … |  |  |  |  |  |  |  |
| 52 | With the frequency of my sexual contacts, I am ... |  |  |  |  |  |  |  |
| 53 | With the frequency with which my spouse turns to me physically (caresses, touches), I am … |  |  |  |  |  |  |  |
| 54 | With my sexual reactions, I am … |  |  |  |  |  |  |  |
| 55 | When I think of how freely I can talk about sexual realm, I am … |  |  |  |  |  |  |  |
| 56 | When I think about the extent to which my partner and I harmonize in terms of sexuality, I am … |  |  |  |  |  |  |  |

|  | **Friends, acquaintances, relatives** | **1 very dissatisfied** | **2 dissatisfied** | **3 rather dissatisfied** | **4 neither/nor** | **5 rather satisfied** | **6 satisfied** | **7 very satisfied** |
| --- | --- | --- | --- | --- | --- | --- | --- | --- |
| 57 | When I think of my friends and acquaintances, I am ... |  |  |  |  |  |  |  |
| 58 | With the contact to my relatives, I am … |  |  |  |  |  |  |  |
| 59 | With the contact to my neighbors, I am ... |  |  |  |  |  |  |  |
| 60 | With the help and support of friends and acquaintances, I am … |  |  |  |  |  |  |  |
| 61 | With my outdoor and community activities (club, church, etc.), I am … |  |  |  |  |  |  |  |
| 62 | With my social commitment, I am … |  |  |  |  |  |  |  |
| 63 | When I think of how often I take part in a social activity, I am … |  |  |  |  |  |  |  |

|  | **Apartment** | **1 very dissatisfied** | **2 dissatisfied** | **3 rather dissatisfied** | **4 neither/nor** | **5 rather satisfied** | **6 satisfied** | **7 very satisfied** |
| --- | --- | --- | --- | --- | --- | --- | --- | --- |
| 64 | With the size of my apartment, I am ... |  |  |  |  |  |  |  |
| 65 | With the state of my apartment, I am … |  |  |  |  |  |  |  |
| 66 | With the expenses (rent, or payment) for my apartment, I am ... |  |  |  |  |  |  |  |
| 67 | With the location of my apartment, I am … |  |  |  |  |  |  |  |
| 68 | With the connection to transport, I am … |  |  |  |  |  |  |  |
| 69 | When I think about the level of noise pollution, I am with my apartment … |  |  |  |  |  |  |  |
| 70 | With the standard of my apartment, I am … |  |  |  |  |  |  |  |

Please answer the following questions by ticking the appropriate box:

**Gender**

o male

o female

**Age: ________** years

**Education**

o completed before the last secondary school class

o finished the last secondary school class

o secondary middle school or commercial school without final exam

o secondary middle school or commercial school with final exam

o high school without high school diploma

o high school diploma without subsequent academic education

o high school diploma with an incomplete academic education

o high school diploma with a completed academic education

**Marital status**

o married / stable partnership

o single

o widowed

o divorced / separated

**Household**

o living alone

o living together with spouse / life partner

**Are you working?**

o yes

o yes, helping out in my own business

o housewife / houseman

**Or are you?**

o pupil

o student

o in vocational training

o retirees, retirement

o unemployed

o without job

**Occupational group**

Please tick the current occupation. (Housewives should tick the occupation of the man / housekeeper the occupation of the woman, pensioners the former occupation, respondents in vocational training and non-employed the occupation of breadwinner)

o Owner or managing director of large companies

o free occupation

o small or medium-sized self-employed business person

o independent craftsmen

o executives

o non-executive employees

o official of the higher service

o official of the middle or lower grade service

o farmer

o skilled worker with passed exam

o other workers

Thank you very much for your cooperation!

**FFCv**

**Fragebogen zum Familienalltag mit einem vom Coma vigile
betroffenen Kind**^[[1]](#footnote-1)^

(Questionnaire about everyday family life with a UWS affected child)

Good day. As a part of my doctorate in the field of curative educational psychology, I developed this questionnaire in cooperation with the human sciences faculty of the University of Cologne. It records the situation in which you live with your child. Your child is affected by Unresponsive Wakefulness Syndrome / coma vigile / vegetative state / Apallic syndrome, and that means a special way of life for you. I would like to find out where you draw your strength from and what you need to cope with the situation. Thank you very much for your participation. I have been working with affected families and their children for a long time, I have learned a lot from them and would like to understand them even better.

Example of an item to be answered. Please make a cross in the corresponding line. You can mark one or several answers.

**What do you like to read?**

| 1. Detective stories | x |
| --- | --- |
| 2. Novels |  |
| 3. Specialist literature | x |

The survey is anonymous, consists of four parts and takes about 45 minutes. Please fill out the forms completely: Do not think too much, but just follow your first feeling. There is no right or wrong answers. All data will be evaluated anonymously. Thank you.

**1) In which country do you live?**

| 1. Germany |  |
| --- | --- |
| 2. Austria |  |
| 3. Switzerland |  |

**2) What is your relation to the affected child? ^[[2]](#footnote-2)^**

| 1. Mother |  |
| --- | --- |
| 2. Father |  |
| 3. Sibling |  |
| 4. Grandma |  |
| 5. Grandpa |  |
| 6. Other family member |  |

**3) What is your age?**

|  |
| --- |

**4) What is the age of the child?**

|  |
| --- |

**5) How do you live?**

| 1. In a house |  |
| --- | --- |
| 2. In an apartment |  |
| 3. We moved after the child has become ill |  |
| 4. We rebuilt our home after the child has become ill |  |

**6) How long have you been living with your affected child?**

|  |
| --- |

**7) When did the event happen that led to the illness of your child?** *(six digit entry of month and year)*

|  |
| --- |

**8) What was the trigger for the vegetative state?^[[3]](#footnote-3)^**

| 1. Birth trauma |  |
| --- | --- |
| 2. Accident |  |
| 3. Illness |  |
| 4. Other |  |

**9) What of the following belongs to the affected child?**

| 1. Own room |  |
| --- | --- |
| 2. Nursing bed |  |
| 3. Tracheostomy tube |  |
| 4. Oxygen supply |  |
| 5. Ventilator |  |
| 6. PEG |  |
| 7. Nutritional pump |  |
| 8. Wheelchair |  |
| 9. Various aids |  |

**10) Whom are you supported by? ^[[4]](#footnote-4)^**

| 1. Family doctor |  |
| --- | --- |
| 2. Nursing service |  |
| 3. Social Pediatric Center |  |
| 4. Therapists |  |
| 5. Health insurance |  |
| 6. Long-term care isurance |  |

**11) How many hours a day (on average) do you get help?**

|  |
| --- |

**12) How many hours a night (on average) do you get help?**

|  |
| --- |

**13) How often therapists or doctors come home?^[[5]](#footnote-5)^**

| 1. Once a week |  |
| --- | --- |
| 2. Three time a week |  |
| 3. Five time a week |  |
| 4. Every two weeks |  |

**14) How often do medical emergencies occur?**

| 1. Once a week |  |
| --- | --- |
| 2. Once a year |  |
| 3. Four times a year |  |
| 4. Every three years |  |

**15) How often do you have to go to external appointments?**

| 1. Once a week |  |
| --- | --- |
| 2. Three times a week |  |
| 3. Once a month |  |
| 4. Twice a year |  |

**16) How often are hospital stays planned?**

| 1. Once a month |  |
| --- | --- |
| 2. Once a year |  |
| 3. Twice a year |  |
| 4. Every two years |  |

**17) How often are operations due?^[[6]](#footnote-6)^**

| 1. Once a year |  |
| --- | --- |
| 2. Twice a year |  |
| 3. Four times a year |  |
| 4. Every two years |  |

**18) Does the affected child go to kindergarden or school?**

| 1. Yes |
| --- |
| 2. No |

**19) Does the sibling have problems?** *(Multiple choices possible)^[[7]](#footnote-7)^*

| 1. None |  |
| --- | --- |
| 2. Insomnia |  |
| 3. Wetting |  |
| 4. Fears |  |
| 5. Hyperactivity |  |
| 6. Thoughtful |  |
| 7. School problems |  |
| 8. No friends |  |
| 9. Others |  |

**20) Do you have problems?** *(Multiple choices possible)*

| 1. None |  |
| --- | --- |
| 2. Insomnia |  |
| 3. Stress |  |
| 4. Fears |  |
| 5. Nutritional disorders |  |
| 6. Drugs of pleasure |  |
| 7. Others |  |

**21) Are you worried?** *(Multiple choices possible)^[[8]](#footnote-8)^*

| 1. None |  |
| --- | --- |
| 2. Ioneliness |  |
| 3. Financial hardship |  |
| 4. Disruption |  |
| 5. Problems in the workplace |  |
| 6. No free time |  |
| 7. No hobbies |  |

**22) What do you want?** *(Multiple choices possible)*

| 1. Privacy |  |
| --- | --- |
| 2. Every day family life |  |
| 3. Less staff turnover |  |
| 4. Be taken seriously |  |
| 5. Therapy |  |
| 6. Financial relief |  |
| 7. Administrative relief |  |

**23) What is positive about your care situation?** *(Multiple choices possible)^[[9]](#footnote-9)^*

| 1. Time relief |  |
| --- | --- |
| 2. Receiving a normal everyday life |  |
| 3. Support through discussions |  |
| 4. Reliance on the child's care |  |
| 5. Exchange with qualified personnel |  |
| 6. Create space for hobbies |  |

**24) How did you feel immediately after it happened to your child?** *(Multiple choices possible)*

| 1. Helpless |  |
| --- | --- |
| 2. Deprived of my power |  |
| 3. The floor was torn away from me |  |
| 4. Anxious |  |
| 5. I didn't feel anything anymore |  |
| 6. I didn't know what to do next |  |
| 7. I had to be strong for my family |  |
| 8. I have obtained specialist literature |  |
| 9. I have been well educated and instructed |  |
| 10. I was given pastoral care |  |
| 11. I worked |  |
| 12. I have started therapy |  |
| 13. I took care of my family |  |
| 14. I´ve talked to other parents |  |
| 15. I felt left alone |  |
| 16. I blamed God |  |

**25) What has changed in your life?** *(Multiple choices possible)^[[10]](#footnote-10)^*

| 1. Everything |  |
| --- | --- |
| 2. Nothing |  |
| 3. My work |  |
| 4. My friends |  |
| 5. My family |  |
| 6. My personality |  |
| 7. My enviroment |  |

**26) How would you describe your life today?** *(Multiple choices possible)^[[11]](#footnote-11)^*

| 1. Satisfactory |  |
| --- | --- |
| 2. Hard |  |
| 3. Unhappy |  |
| 4. Desperate |  |
| 5. Happy |  |
| 6. Familiar |  |
| 7. Consolidated |  |

**Thank you for your patience and your openness.**

1. Reprinting and any form of reproduction prohibited. Christiane Chadasch, born Klapheck, holds the rights. [↑](#footnote-ref-1)
2. [↑](#footnote-ref-2)
3. [↑](#footnote-ref-3)
4. [↑](#footnote-ref-4)
5. [↑](#footnote-ref-5)
6. [↑](#footnote-ref-6)
7. [↑](#footnote-ref-7)
8. [↑](#footnote-ref-8)
9. [↑](#footnote-ref-9)
10. [↑](#footnote-ref-10)
11. [↑](#footnote-ref-11)
