# Supplementary material for: Life satisfaction in families with a child in an Unresponsive Wakefulness Syndrome
Source: BMC Pediatr. 2021 Mar 8;21:116. doi: 10.1186/s12887-021-02549-8 (PMC7938537; doi:10.1186/s12887-021-02549-8)
Supplement: Supplementary file 2 — Additional file 2. Supplement 2. Frequency tables. [file 12887_2021_2549_MOESM2_ESM.docx]

**Supplement 2. Frequency tables**

| *Homeland* | | | | | |
| --- | --- | --- | --- | --- | --- |
| Group | | | Frequency | Percent | Valid Percent |
| 0 Control group | Valid | Germany | 26 | 100,0 | 100,0 |
| 1 Affected group | Valid | Germany | 21 | 80,8 | 80,8 |
|  |  | Austria | 5 | 19,2 | 19,2 |
|  |  | Total | 26 | 100,0 | 100,0 |
|  |  |  |  |  |  |
| *Participant* | | | | | |
| Group | | | Frequency | Percent | Valid Percent |
| 0 Control group | Valid | Mother | 23 | 88,5 | 88,5 |
|  |  | Father | 3 | 11,5 | 11,5 |
|  |  | Total | 26 | 100,0 | 100,0 |
| 1 Affected group | Valid | Mother | 25 | 96,2 | 96,2 |
|  |  | Father | 1 | 3,8 | 3,8 |
|  |  | Total | 26 | 100,0 | 100,0 |
|  |  |  |  |  |  |
| *Age of the participant* | | | | | |
| Group | | | Frequency | Percent | Cumulative Percent |
| 0 Control group | Valid | 29 | 1 | 3,8 | 3,8 |
|  |  | 35 | 1 | 3,8 | 7,7 |
|  |  | 36 | 1 | 3,8 | 11,5 |
|  |  | 38 | 3 | 11,5 | 23,1 |
|  |  | 39 | 3 | 11,5 | 34,6 |
|  |  | 40 | 1 | 3,8 | 38,5 |
|  |  | 41 | 2 | 7,7 | 46,2 |
|  |  | 42 | 6 | 23,1 | 69,2 |
|  |  | 43 | 1 | 3,8 | 73,1 |
|  |  | 44 | 1 | 3,8 | 76,9 |
|  |  | 48 | 1 | 3,8 | 80,8 |
|  |  | 49 | 1 | 3,8 | 84,6 |
|  |  | 52 | 1 | 3,8 | 88,5 |
|  |  | 53 | 1 | 3,8 | 92,3 |
|  |  | 56 | 1 | 3,8 | 96,2 |
|  |  | 61 | 1 | 3,8 | 100,0 |
|  |  | Total | 26 | 100,0 |  |
| 1 Affected group | Valid | 32 | 1 | 3,8 | 3,8 |
|  |  | 34 | 2 | 7,7 | 11,5 |
|  |  | 38 | 4 | 15,4 | 26,9 |
|  |  | 40 | 3 | 11,5 | 38,5 |
|  |  | 41 | 3 | 11,5 | 50,0 |
|  |  | 42 | 2 | 7,7 | 57,7 |
|  |  | 43 | 2 | 7,7 | 65,4 |
|  |  | 44 | 3 | 11,5 | 76,9 |
|  |  | 46 | 1 | 3,8 | 80,8 |
|  |  | 49 | 1 | 3,8 | 84,6 |
|  |  | 50 | 1 | 3,8 | 88,5 |
|  |  | 52 | 1 | 3,8 | 92,3 |
|  |  | 54 | 1 | 3,8 | 96,2 |
|  |  | 58 | 1 | 3,8 | 100,0 |
|  |  | Total | 26 | 100,0 |  |
|  |  |  |  |  |  |
| *Age of the child* | | | | | |
| Group | | | Frequency | Percent | Cumulative Percent |
| 0 Control group | Valid | 2 | 1 | 3,8 | 3,8 |
|  |  | 3 | 1 | 3,8 | 7,7 |
|  |  | 4 | 1 | 3,8 | 11,5 |
|  |  | 5 | 1 | 3,8 | 15,4 |
|  |  | 6 | 3 | 11,5 | 26,9 |
|  |  | 7 | 4 | 15,4 | 42,3 |
|  |  | 8 | 1 | 3,8 | 46,2 |
|  |  | 9 | 1 | 3,8 | 50,0 |
|  |  | 10 | 1 | 3,8 | 53,8 |
|  |  | 11 | 4 | 15,4 | 69,2 |
|  |  | 14 | 1 | 3,8 | 73,1 |
|  |  | 15 | 1 | 3,8 | 76,9 |
|  |  | 16 | 1 | 3,8 | 80,8 |
|  |  | 19 | 2 | 7,7 | 88,5 |
|  |  | 20 | 1 | 3,8 | 92,3 |
|  |  | 23 | 1 | 3,8 | 96,2 |
|  |  | 24 | 1 | 3,8 | 100,0 |
|  |  | Total | 26 | 100,0 |  |
| 1 Affected group | Valid | 2 | 1 | 3,8 | 3,8 |
|  |  | 3 | 1 | 3,8 | 7,7 |
|  |  | 4 | 1 | 3,8 | 11,5 |
|  |  | 6 | 4 | 15,4 | 26,9 |
|  |  | 7 | 3 | 11,5 | 38,5 |
|  |  | 8 | 1 | 3,8 | 42,3 |
|  |  | 9 | 1 | 3,8 | 46,2 |
|  |  | 10 | 2 | 7,7 | 53,8 |
|  |  | 11 | 3 | 11,5 | 65,4 |
|  |  | 14 | 1 | 3,8 | 69,2 |
|  |  | 15 | 2 | 7,7 | 76,9 |
|  |  | 16 | 1 | 3,8 | 80,8 |
|  |  | 19 | 2 | 7,7 | 88,5 |
|  |  | 20 | 1 | 3,8 | 92,3 |
|  |  | 23 | 1 | 3,8 | 96,2 |
|  |  | 24 | 1 | 3,8 | 100,0 |
|  |  | Total | 26 | 100,0 |  |
|  |  |  |  |  |  |
| *Living condition* | | | | | |
| Group | | | Frequency | Percent | Cumulative Percent |
| 0 Control group | Missing | System | 26 | 100,0 |  |
| 1 Affected group | Valid | House | 17 | 65,4 | 65,4 |
|  |  | Apartment | 9 | 34,6 | 100,0 |
|  |  | Total | 26 | 100,0 |  |
|  |  |  |  |  |  |
| *Was moving necessary?* | | | | | |
| Group | | | Frequency | Percent | Cumulative Percent |
| 0 Control group | Missing | System | 26 | 100,0 |  |
| 1 Affected group | Valid | no | 11 | 42,3 | 42,3 |
|  |  | yes | 10 | 38,5 | 80,8 |
|  |  | Missing | 5 | 19,2 | 100,0 |
|  |  | Total | 26 | 100,0 |  |
|  |  |  |  |  |  |
| *Since when living together* | | | | | |
| Group | | | Frequency | Percent | Cumulative Percent |
| 0 Control group | Missing | System | 26 | 100,0 |  |
| 1 Affected group | Valid | 0 | 1 | 3,8 | 3,8 |
|  |  | 1 | 1 | 3,8 | 7,7 |
|  |  | 3 | 2 | 7,7 | 15,4 |
|  |  | 4 | 3 | 11,5 | 26,9 |
|  |  | 6 | 5 | 19,2 | 46,2 |
|  |  | 7 | 2 | 7,7 | 53,8 |
|  |  | 8 | 1 | 3,8 | 57,7 |
|  |  | 10 | 2 | 7,7 | 65,4 |
|  |  | 11 | 1 | 3,8 | 69,2 |
|  |  | 12 | 1 | 3,8 | 73,1 |
|  |  | 15 | 2 | 7,7 | 80,8 |
|  |  | 16 | 1 | 3,8 | 84,6 |
|  |  | 19 | 2 | 7,7 | 92,3 |
|  |  | 20 | 1 | 3,8 | 96,2 |
|  |  | 24 | 1 | 3,8 | 100,0 |
|  |  | Total | 26 | 100,0 |  |
|  |  |  |  |  |  |
| *Age at the time of injury* | | | | | |
| Group | | | Frequency | Percent | Cumulative Percent |
| 0 Control group | Missing | System | 26 | 100,0 |  |
| 1 Affected group | Valid | 0 | 5 | 19,2 | 19,2 |
|  |  | 1 | 5 | 19,2 | 38,5 |
|  |  | 2 | 3 | 11,5 | 50,0 |
|  |  | 3 | 5 | 19,2 | 69,2 |
|  |  | 4 | 1 | 3,8 | 73,1 |
|  |  | 5 | 1 | 3,8 | 76,9 |
|  |  | 6 | 1 | 3,8 | 80,8 |
|  |  | 8 | 1 | 3,8 | 84,6 |
|  |  | 11 | 1 | 3,8 | 88,5 |
|  |  | 14 | 1 | 3,8 | 92,3 |
|  |  | 16 | 1 | 3,8 | 96,2 |
|  |  | 17 | 1 | 3,8 | 100,0 |
|  |  | Total | 26 | 100,0 |  |
|  |  |  |  |  |  |
| *Character of the injury* | | | | | |
| Group | | | Frequency | Percent | Cumulative Percent |
| 0 Control group | Missing | System | 26 | 100,0 |  |
| 1 Affected group | Valid | Birth trauma | 1 | 3,8 | 3,8 |
|  |  | traumatic brain injury | 16 | 61,5 | 65,4 |
|  |  | illness | 7 | 26,9 | 92,3 |
|  |  | others | 2 | 7,7 | 100,0 |
|  |  | Total | 26 | 100,0 |  |
|  |  |  |  |  |  |
| *Does the child has own room?* | | | | | |
| Group | | | Frequency | Percent | Cumulative Percent |
| 0 Control group | Missing | System | 26 | 100,0 |  |
| 1 Affected group | Valid | no | 2 | 7,7 | 7,7 |
|  |  | yes | 24 | 92,3 | 100,0 |
|  |  | Total | 26 | 100,0 |  |
|  |  |  |  |  |  |
| *Functional bed* | | | | | |
| Group | | | Frequency | Percent | Cumulative Percent |
| 0 Control group | Missing | System | 26 | 100,0 |  |
| 1 Affected group | Valid | no | 1 | 3,8 | 3,8 |
|  |  | yes | 25 | 96,2 | 100,0 |
|  |  | Total | 26 | 100,0 |  |
|  |  |  |  |  |  |
| *tracheal cannula* | | | | | |
| Group | | | Frequency | Percent | Cumulative Percent |
| 0 Control group | Missing | System | 26 | 100,0 |  |
| 1 Affected group | Valid | no | 21 | 80,8 | 80,8 |
|  |  | yes | 5 | 19,2 | 100,0 |
|  |  | Total | 26 | 100,0 |  |
|  |  |  |  |  |  |
| *oxygen support, at least short time* | | | | | |
| Group | | | Frequency | Percent | Cumulative Percent |
| 0 Control group | Missing | System | 26 | 100,0 |  |
| 1 Affected group | Valid | no | 17 | 65,4 | 65,4 |
|  |  | yes | 9 | 34,6 | 100,0 |
|  |  | Total | 26 | 100,0 |  |
|  |  |  |  |  |  |
| *ventilation, at least short time* | | | | | |
| Group | | | Frequency | Percent | Cumulative Percent |
| 0 Control group | Missing | System | 26 | 100,0 |  |
| 1 Affected group | Valid | no | 22 | 84,6 | 84,6 |
|  |  | yes | 4 | 15,4 | 100,0 |
|  |  | Total | 26 | 100,0 |  |
|  |  |  |  |  |  |
| *PEG* | | | | | |
| Group | | | Frequency | Percent | Cumulative Percent |
| 0 Control group | Missing | System | 26 | 100,0 |  |
| 1 Affected group | Valid | no | 2 | 7,7 | 7,7 |
|  |  | yes | 24 | 92,3 | 100,0 |
|  |  | Total | 26 | 100,0 |  |
|  |  |  |  |  |  |
| *Nutrition pump* | | | | | |
| Group | | | Frequency | Percent | Cumulative Percent |
| 0 Control group | Missing | System | 26 | 100,0 |  |
| 1 Affected group | Valid | no | 9 | 34,6 | 34,6 |
|  |  | yes | 17 | 65,4 | 100,0 |
|  |  | Total | 26 | 100,0 |  |
|  |  |  |  |  |  |
| *Wheelchair* | | | | | |
| Group | | | Frequency | Percent | Cumulative Percent |
| 0 Control group | Missing | System | 26 | 100,0 |  |
| 1 Affected group | Valid | no | 3 | 11,5 | 11,5 |
|  |  | yes | 23 | 88,5 | 100,0 |
|  |  | Total | 26 | 100,0 |  |
|  |  |  |  |  |  |
| *Other supporting devices* | | | | | |
| Group | | | Frequency | Percent | Cumulative Percent |
| 0 Control group | Missing | System | 26 | 100,0 |  |
| 1 Affected group | Valid | yes | 26 | 100,0 | 100,0 |
|  |  |  |  |  |  |
| *Support from family doctor* | | | | | |
| Group | | | Frequency | Percent | Cumulative Percent |
| 0 Control group | Missing | System | 26 | 100,0 |  |
| 1 Affected group | Valid | no | 6 | 23,1 | 23,1 |
|  |  | yes | 20 | 76,9 | 100,0 |
|  |  | Total | 26 | 100,0 |  |
|  |  |  |  |  |  |
| *Support from nursing personnel* | | | | | |
| Group | | | Frequency | Percent | Cumulative Percent |
| 0 Control group | Missing | System | 26 | 100,0 |  |
| 1 Affected group | Valid | no | 10 | 38,5 | 38,5 |
|  |  | yes | 16 | 61,5 | 100,0 |
|  |  | Total | 26 | 100,0 |  |
|  |  |  |  |  |  |
| *SPZ* | | | | | |
| Group | | | Frequency | Percent | Cumulative Percent |
| 0 Control group | Missing | System | 26 | 100,0 |  |
| 1 Affected group | Valid | no | 18 | 69,2 | 69,2 |
|  |  | yes | 8 | 30,8 | 100,0 |
|  |  | Total | 26 | 100,0 |  |
|  |  |  |  |  |  |
| *Other medical professionals (physical therapy, speech therapy, etc.)* | | | | | |
| Group | | | Frequency | Percent | Cumulative Percent |
| 0 Control group | Missing | System | 26 | 100,0 |  |
| 1 Affected group | Valid | no | 2 | 7,7 | 7,7 |
|  |  | yes | 24 | 92,3 | 100,0 |
|  |  | Total | 26 | 100,0 |  |
|  |  |  |  |  |  |
| *Support from health insurance* | | | | | |
| Group | | | Frequency | Percent | Cumulative Percent |
| 0 Control group | Missing | System | 26 | 100,0 |  |
| 1 Affected group | Valid | no | 8 | 30,8 | 30,8 |
|  |  | yes | 18 | 69,2 | 100,0 |
|  |  | Total | 26 | 100,0 |  |
|  |  |  |  |  |  |
| *Support from nursing care insurance* | | | | | |
| Group | | | Frequency | Percent | Cumulative Percent |
| 0 Control group | Missing | System | 26 | 100,0 |  |
| 1 Affected group | Valid | no | 12 | 46,2 | 46,2 |
|  |  | yes | 14 | 53,8 | 100,0 |
|  |  | Total | 26 | 100,0 |  |
|  |  |  |  |  |  |
| *Support during daytime (hours per day)* | | | | | |
| Group | | | Frequency | Percent | Cumulative Percent |
| 0 Control group | Missing | System | 26 | 100,0 |  |
| 1 Affected group | Valid | no | 5 | 19,2 | 19,2 |
|  |  | <1 | 1 | 3,8 | 23,1 |
|  |  | 1 | 3 | 11,5 | 34,6 |
|  |  | 2 | 4 | 15,4 | 50,0 |
|  |  | 3 | 1 | 3,8 | 53,8 |
|  |  | 6 | 2 | 7,7 | 61,5 |
|  |  | 7 | 2 | 7,7 | 69,2 |
|  |  | 8 | 3 | 11,5 | 80,8 |
|  |  | 11 | 1 | 3,8 | 84,6 |
|  |  | 12 | 4 | 15,4 | 100,0 |
|  |  | Total | 26 | 100,0 |  |
|  |  |  |  |  |  |
| *Support during nighttime (hours per day)* | | | | | |
| Group | | | Frequency | Percent | Cumulative Percent |
| 0 Control group | Missing | System | 26 | 100,0 |  |
| 1 Affected group | Valid | no | 19 | 73,1 | 73,1 |
|  |  | 1 | 1 | 3,8 | 76,9 |
|  |  | 8 | 1 | 3,8 | 80,8 |
|  |  | 12 | 5 | 19,2 | 100,0 |
|  |  | Total | 26 | 100,0 |  |
|  |  |  |  |  |  |
| *Frequency of visits of doctors or therapists* | | | | | |
| Group | | | Frequency | Percent | Cumulative Percent |
| 0 Control group | Missing | System | 26 | 100,0 |  |
| 1 Affected group | Valid | no | 2 | 7,7 | 7,7 |
|  |  | one per week or less | 7 | 26,9 | 34,6 |
|  |  | three times per week | 4 | 15,4 | 50,0 |
|  |  | five time per week | 13 | 50,0 | 100,0 |
|  |  | Total | 26 | 100,0 |  |
|  |  |  |  |  |  |
| *Frequency of emergencies* | | | | | |
| Group | | | Frequency | Percent | Cumulative Percent |
| 0 Control group | Missing | System | 26 | 100,0 |  |
| 1 Affected group | Valid | weekly | 5 | 19,2 | 22,7 |
|  |  | about 4 times / year | 6 | 23,1 | 50,0 |
|  |  | about 1 time / year | 7 | 26,9 | 81,8 |
|  |  | about once in 3 years | 4 | 15,4 | 100,0 |
|  |  | Total | 22 | 84,6 |  |
|  | Missing | 0 | 4 | 15,4 |  |
|  | Total | | 26 | 100,0 |  |
|  |  |  |  |  |  |
| *Frequency of external appointments* | | | | | |
| Group | | | Frequency | Percent | Cumulative Percent |
| 0 Control group | Missing | System | 26 | 100,0 |  |
| 1 Affected group | Valid | about 3 per week | 5 | 19,2 | 19,2 |
|  |  | about 1 per week | 7 | 26,9 | 46,2 |
|  |  | about 1 per month | 8 | 30,8 | 76,9 |
|  |  | about 1 per year or less | 6 | 23,1 | 100,0 |
|  |  | Total | 26 | 100,0 |  |
|  |  |  |  |  |  |
| *Frequency of hospital stays* | | | | | |
| Group | | | Frequency | Percent | Cumulative Percent |
| 0 Control group | Missing | System | 26 | 100,0 |  |
| 1 Affected group | Valid | no | 8 | 30,8 | 30,8 |
|  |  | about 1 per month | 4 | 15,4 | 46,2 |
|  |  | about 2 per year | 5 | 19,2 | 65,4 |
|  |  | about 1 per year | 5 | 19,2 | 84,6 |
|  |  | less than 1 per year | 4 | 15,4 | 100,0 |
|  |  | Total | 26 | 100,0 |  |
|  |  |  |  |  |  |
| *frequency of surgical interventions* | | | | | |
| Group | | | Frequency | Percent | Cumulative Percent |
| 0 Control group | Missing | System | 26 | 100,0 |  |
| 1 Affected group | Valid | no | 9 | 34,6 | 34,6 |
|  |  | 1 | 8 | 30,8 | 65,4 |
|  |  | 3 | 1 | 3,8 | 69,2 |
|  |  | 4 | 7 | 26,9 | 96,2 |
|  |  | 5 | 1 | 3,8 | 100,0 |
|  |  | Total | 26 | 100,0 |  |
|  |  |  |  |  |  |
| *Visit of other children institutions* | | | | | |
| Group | | | Frequency | Percent | Cumulative Percent |
| 0 Control group | Missing | System | 26 | 100,0 |  |
| 1 Affected group | Valid | no | 5 | 19,2 | 19,2 |
|  |  | yes | 21 | 80,8 | 100,0 |
|  |  | Total | 26 | 100,0 |  |
|  |  |  |  |  |  |
| *Sibling have no problems* | | | | | |
| Group | | | Frequency | Percent | Cumulative Percent |
| 0 Control group | Missing | System | 26 | 100,0 |  |
| 1 Affected group | Valid | agree | 15 | 57,7 | 57,7 |
|  |  | disagree | 11 | 42,3 | 100,0 |
|  |  | Total | 26 | 100,0 |  |
|  |  |  |  |  |  |
| *sleep disorders* | | | | | |
| Group | | | Frequency | Percent | Cumulative Percent |
| 0 Control group | Missing | System | 26 | 100,0 |  |
| 1 Affected group | Valid | no | 26 | 100,0 | 100,0 |
|  |  |  |  |  |  |
| *wetting in bed* | | | | | |
| Group | | | Frequency | Percent | Cumulative Percent |
| 0 Control group | Missing | System | 26 | 100,0 |  |
| 1 Affected group | Valid | no | 25 | 96,2 | 96,2 |
|  |  | yes | 1 | 3,8 | 100,0 |
|  |  | Total | 26 | 100,0 |  |
|  |  |  |  |  |  |
| *Anxiety* | | | | | |
| Group | | | Frequency | Percent | Cumulative Percent |
| 0 Control group | Missing | System | 26 | 100,0 |  |
| 1 Affected group | Valid | no | 23 | 88,5 | 88,5 |
|  |  | yes | 3 | 11,5 | 100,0 |
|  |  | Total | 26 | 100,0 |  |
|  |  |  |  |  |  |
| *Hyperactivity* | | | | | |
| Group | | | Frequency | Percent | Cumulative Percent |
| 0 Control group | Missing | System | 26 | 100,0 |  |
| 1 Affected group | Valid | no | 23 | 88,5 | 88,5 |
|  |  | yes | 3 | 11,5 | 100,0 |
|  |  | Total | 26 | 100,0 |  |
|  |  |  |  |  |  |
| *Rumination* | | | | | |
| Group | | | Frequency | Percent | Cumulative Percent |
| 0 Control group | Missing | System | 26 | 100,0 |  |
| 1 Affected group | Valid | no | 19 | 73,1 | 73,1 |
|  |  | yes | 7 | 26,9 | 100,0 |
|  |  | Total | 26 | 100,0 |  |
|  |  |  |  |  |  |
| *School problems* | | | | | |
| Group | | | Frequency | Percent | Cumulative Percent |
| 0 Control group | Missing | System | 26 | 100,0 |  |
| 1 Affected group | Valid | no | 23 | 88,5 | 88,5 |
|  |  | yes | 3 | 11,5 | 100,0 |
|  |  | Total | 26 | 100,0 |  |
|  |  |  |  |  |  |
| *Problems with social contacts* | | | | | |
| Group | | | Frequency | Percent | Cumulative Percent |
| 0 Control group | Missing | System | 26 | 100,0 |  |
| 1 Affected group | Valid | no | 25 | 96,2 | 96,2 |
|  |  | yes | 1 | 3,8 | 100,0 |
|  |  | Total | 26 | 100,0 |  |
|  |  |  |  |  |  |
| *Other problems* | | | | | |
| Group | | | Frequency | Percent | Cumulative Percent |
| 0 Control group | Missing | System | 26 | 100,0 |  |
| 1 Affected group | Valid | no | 24 | 92,3 | 92,3 |
|  |  | yes | 2 | 7,7 | 100,0 |
|  |  | Total | 26 | 100,0 |  |
|  |  |  |  |  |  |
| *Parents' Problems: None* | | | | | |
| Group | | | Frequency | Percent | Cumulative Percent |
| 0 Control group | Missing | System | 26 | 100,0 |  |
| 1 Affected group | Valid | disagree | 23 | 88,5 | 88,5 |
|  |  | agree | 3 | 11,5 | 100,0 |
|  |  | Total | 26 | 100,0 |  |
|  |  |  |  |  |  |
| *sleep disorders* | | | | | |
| Group | | | Frequency | Percent | Cumulative Percent |
| 0 Control group | Missing | System | 26 | 100,0 |  |
| 1 Affected group | Valid | no | 14 | 53,8 | 53,8 |
|  |  | yes | 12 | 46,2 | 100,0 |
|  |  | Total | 26 | 100,0 |  |
|  |  |  |  |  |  |
| *Stress* | | | | | |
| Group | | | Frequency | Percent | Cumulative Percent |
| 0 Control group | Missing | System | 26 | 100,0 |  |
| 1 Affected group | Valid | no | 10 | 38,5 | 38,5 |
|  |  | yes | 16 | 61,5 | 100,0 |
|  |  | Total | 26 | 100,0 |  |
|  |  |  |  |  |  |
| *Anxiety* | | | | | |
| Group | | | Frequency | Percent | Cumulative Percent |
| 0 Control group | Missing | System | 26 | 100,0 |  |
| 1 Affected group | Valid | no | 12 | 46,2 | 46,2 |
|  |  | yes | 14 | 53,8 | 100,0 |
|  |  | Total | 26 | 100,0 |  |
|  |  |  |  |  |  |
| *Eating* | | | | | |
| Group | | | Frequency | Percent | Cumulative Percent |
| 0 Control group | Missing | System | 26 | 100,0 |  |
| 1 Affected group | Valid | no | 22 | 84,6 | 84,6 |
|  |  | yes | 4 | 15,4 | 100,0 |
|  |  | Total | 26 | 100,0 |  |
|  |  |  |  |  |  |
| *Eating disorders* | | | | | |
| Group | | | Frequency | Percent | Cumulative Percent |
| 0 Control group | Missing | System | 26 | 100,0 |  |
| 1 Affected group | Valid | no | 23 | 88,5 | 88,5 |
|  |  | yes | 3 | 11,5 | 100,0 |
|  |  | Total | 26 | 100,0 |  |
|  |  |  |  |  |  |
| *Drugs (including alcohol tobacco, etc.)* | | | | | |
| Group | | | Frequency | Percent | Cumulative Percent |
| 0 Control group | Missing | System | 26 | 100,0 |  |
| 1 Affected group | Valid | no | 18 | 69,2 | 69,2 |
|  |  | yes | 8 | 30,8 | 100,0 |
|  |  | Total | 26 | 100,0 |  |
|  |  |  |  |  |  |
| *Other problems* | | | | | |
| Group | | | Frequency | Percent | Cumulative Percent |
| 0 Control group | Missing | System | 26 | 100,0 |  |
| 1 Affected group | Valid | no | 24 | 92,3 | 92,3 |
|  |  | yes | 2 | 7,7 | 100,0 |
|  |  | Total | 26 | 100,0 |  |
|  |  |  |  |  |  |
| *Situational worries: None* | | | | | |
| Group | | | Frequency | Percent | Cumulative Percent |
| 0 Control group | Missing | System | 26 | 100,0 |  |
| 1 Affected group | Valid | disagree | 21 | 80,8 | 80,8 |
|  |  | agree | 5 | 19,2 | 100,0 |
|  |  | Total | 26 | 100,0 |  |
|  |  |  |  |  |  |
| *Loneliness* | | | | | |
| Group | | | Frequency | Percent | Cumulative Percent |
| 0 Control group | Missing | System | 26 | 100,0 |  |
| 1 Affected group | Valid | no | 19 | 73,1 | 73,1 |
|  |  | yes | 7 | 26,9 | 100,0 |
|  |  | Total | 26 | 100,0 |  |
|  |  |  |  |  |  |
| *Financial problems* | | | | | |
| Group | | | Frequency | Percent | Cumulative Percent |
| 0 Control group | Missing | System | 26 | 100,0 |  |
| 1 Affected group | Valid | no | 12 | 46,2 | 46,2 |
|  |  | yes | 14 | 53,8 | 100,0 |
|  |  | Total | 26 | 100,0 |  |
|  |  |  |  |  |  |
| *Disruption* | | | | | |
| Group | | | Frequency | Percent | Cumulative Percent |
| 0 Control group | Missing | System | 26 | 100,0 |  |
| 1 Affected group | Valid | no | 12 | 46,2 | 46,2 |
|  |  | yes | 14 | 53,8 | 100,0 |
|  |  | Total | 26 | 100,0 |  |
|  |  |  |  |  |  |
| *Job problems* | | | | | |
| Group | | | Frequency | Percent | Cumulative Percent |
| 0 Control group | Missing | System | 26 | 100,0 |  |
| 1 Affected group | Valid | no | 21 | 80,8 | 80,8 |
|  |  | yes | 5 | 19,2 | 100,0 |
|  |  | Total | 26 | 100,0 |  |
|  |  |  |  |  |  |
| *Lack of leisure* | | | | | |
| Group | | | Frequency | Percent | Cumulative Percent |
| 0 Control group | Missing | System | 26 | 100,0 |  |
| 1 Affected group | Valid | no | 8 | 30,8 | 30,8 |
|  |  | yes | 18 | 69,2 | 100,0 |
|  |  | Total | 26 | 100,0 |  |
|  |  |  |  |  |  |
| *Lack of hobbies* | | | | | |
| Group | | | Frequency | Percent | Cumulative Percent |
| 0 Control group | Missing | System | 26 | 100,0 |  |
| 1 Affected group | Valid | no | 16 | 61,5 | 61,5 |
|  |  | yes | 10 | 38,5 | 100,0 |
|  |  | Total | 26 | 100,0 |  |
|  |  |  |  |  |  |
| *Lack of privacy* | | | | | |
| Group | | | Frequency | Percent | Cumulative Percent |
| 0 Control group | Missing | System | 26 | 100,0 |  |
| 1 Affected group | Valid | no | 14 | 53,8 | 53,8 |
|  |  | yes | 11 | 42,3 | 96,2 |
|  |  | Missing | 1 | 3,8 | 100,0 |
|  |  | Total | 26 | 100,0 |  |
|  |  |  |  |  |  |
| *Family routine* | | | | | |
| Group | | | Frequency | Percent | Cumulative Percent |
| 0 Control group | Missing | System | 26 | 100,0 |  |
| 1 Affected group | Valid | no | 11 | 42,3 | 42,3 |
|  |  | yes | 15 | 57,7 | 100,0 |
|  |  | Total | 26 | 100,0 |  |
|  |  |  |  |  |  |
| *Personnel turnover* | | | | | |
| Group | | | Frequency | Percent | Cumulative Percent |
| 0 Control group | Missing | System | 26 | 100,0 |  |
| 1 Affected group | Valid | no | 18 | 69,2 | 69,2 |
|  |  | yes | 8 | 30,8 | 100,0 |
|  |  | Total | 26 | 100,0 |  |
|  |  |  |  |  |  |
| *Acceptance* | | | | | |
| Group | | | Frequency | Percent | Cumulative Percent |
| 0 Control group | Missing | System | 26 | 100,0 |  |
| 1 Affected group | Valid | no | 14 | 53,8 | 53,8 |
|  |  | yes | 12 | 46,2 | 100,0 |
|  |  | Total | 26 | 100,0 |  |
|  |  |  |  |  |  |
| *Therapy* | | | | | |
| Group | | | Frequency | Percent | Cumulative Percent |
| 0 Control group | Missing | System | 26 | 100,0 |  |
| 1 Affected group | Valid | no | 17 | 65,4 | 65,4 |
|  |  | yes | 9 | 34,6 | 100,0 |
|  |  | Total | 26 | 100,0 |  |
|  |  |  |  |  |  |
| *Financial support* | | | | | |
| Group | | | Frequency | Percent | Cumulative Percent |
| 0 Control group | Missing | System | 26 | 100,0 |  |
| 1 Affected group | Valid | no | 10 | 38,5 | 38,5 |
|  |  | yes | 16 | 61,5 | 100,0 |
|  |  | Total | 26 | 100,0 |  |
|  |  |  |  |  |  |
| *Administrative support* | | | | | |
| Group | | | Frequency | Percent | Cumulative Percent |
| 0 Control group | Missing | System | 26 | 100,0 |  |
| 1 Affected group | Valid | no | 15 | 57,7 | 57,7 |
|  |  | yes | 11 | 42,3 | 100,0 |
|  |  | Total | 26 | 100,0 |  |
|  |  |  |  |  |  |
| *Logistic support* | | | | | |
| Group | | | Frequency | Percent | Cumulative Percent |
| 0 Control group | Missing | System | 26 | 100,0 |  |
| 1 Affected group | Valid | no | 11 | 42,3 | 42,3 |
|  |  | yes | 15 | 57,7 | 100,0 |
|  |  | Total | 26 | 100,0 |  |
|  |  |  |  |  |  |
| *Normal daily routine* | | | | | |
| Group | | | Frequency | Percent | Cumulative Percent |
| 0 Control group | Missing | System | 26 | 100,0 |  |
| 1 Affected group | Valid | no | 15 | 57,7 | 57,7 |
|  |  | yes | 11 | 42,3 | 100,0 |
|  |  | Total | 26 | 100,0 |  |
|  |  |  |  |  |  |
| *Support through conversations* | | | | | |
| Group | | | Frequency | Percent | Cumulative Percent |
| 0 Control group | Missing | System | 26 | 100,0 |  |
| 1 Affected group | Valid | no | 18 | 69,2 | 69,2 |
|  |  | yes | 8 | 30,8 | 100,0 |
|  |  | Total | 26 | 100,0 |  |
|  |  |  |  |  |  |
| *Trust that the child is cared for correctly* | | | | | |
| Group | | | Frequency | Percent | Cumulative Percent |
| 0 Control group | Missing | System | 26 | 100,0 |  |
| 1 Affected group | Valid | no | 14 | 53,8 | 53,8 |
|  |  | yes | 12 | 46,2 | 100,0 |
|  |  | Total | 26 | 100,0 |  |
|  |  |  |  |  |  |
| *Contact with experts* | | | | | |
| Group | | | Frequency | Percent | Cumulative Percent |
| 0 Control group | Missing | System | 26 | 100,0 |  |
| 1 Affected group | Valid | no | 14 | 53,8 | 53,8 |
|  |  | yes | 12 | 46,2 | 100,0 |
|  |  | Total | 26 | 100,0 |  |
|  |  |  |  |  |  |
| *Free time for hobbies* | | | | | |
| Group | | | Frequency | Percent | Cumulative Percent |
| 0 Control group | Missing | System | 26 | 100,0 |  |
| 1 Affected group | Valid | no | 18 | 69,2 | 69,2 |
|  |  | yes | 8 | 30,8 | 100,0 |
|  |  | Total | 26 | 100,0 |  |
|  |  |  |  |  |  |
| *Feelings immediately after the event: helplessness* | | | | | |
| Group | | | Frequency | Percent | Cumulative Percent |
| 0 Control group | Missing | System | 26 | 100,0 |  |
| 1 Affected group | Valid | no | 10 | 38,5 | 38,5 |
|  |  | yes | 16 | 61,5 | 100,0 |
|  |  | Total | 26 | 100,0 |  |
|  |  |  |  |  |  |
| *Power loss* | | | | | |
| Group | | | Frequency | Percent | Cumulative Percent |
| 0 Control group | Missing | System | 26 | 100,0 |  |
| 1 Affected group | Valid | no | 16 | 61,5 | 61,5 |
|  |  | yes | 10 | 38,5 | 100,0 |
|  |  | Total | 26 | 100,0 |  |
|  |  |  |  |  |  |
| *Fathomlessness* | | | | | |
| Group | | | Frequency | Percent | Cumulative Percent |
| 0 Control group | Missing | System | 26 | 100,0 |  |
| 1 Affected group | Valid | no | 6 | 23,1 | 23,1 |
|  |  | yes | 20 | 76,9 | 100,0 |
|  |  | Total | 26 | 100,0 |  |
|  |  |  |  |  |  |
| *Anxiety* | | | | | |
| Group | | | Frequency | Percent | Cumulative Percent |
| 0 Control group | Missing | System | 26 | 100,0 |  |
| 1 Affected group | Valid | no | 13 | 50,0 | 50,0 |
|  |  | yes | 13 | 50,0 | 100,0 |
|  |  | Total | 26 | 100,0 |  |
|  |  |  |  |  |  |
| *Loss of feelings* | | | | | |
| Group | | | Frequency | Percent | Cumulative Percent |
| 0 Control group | Missing | System | 26 | 100,0 |  |
| 1 Affected group | Valid | no | 21 | 80,8 | 80,8 |
|  |  | yes | 5 | 19,2 | 100,0 |
|  |  | Total | 26 | 100,0 |  |
|  |  |  |  |  |  |
| *Loss of perspective* | | | | | |
| Group | | | Frequency | Percent | Cumulative Percent |
| 0 Control group | Missing | System | 26 | 100,0 |  |
| 1 Affected group | Valid | no | 10 | 38,5 | 38,5 |
|  |  | yes | 16 | 61,5 | 100,0 |
|  |  | Total | 26 | 100,0 |  |
|  |  |  |  |  |  |
| *Need to be strong for the family* | | | | | |
| Group | | | Frequency | Percent | Cumulative Percent |
| 0 Control group | Missing | System | 26 | 100,0 |  |
| 1 Affected group | Valid | no | 8 | 30,8 | 30,8 |
|  |  | yes | 18 | 69,2 | 100,0 |
|  |  | Total | 26 | 100,0 |  |
|  |  |  |  |  |  |
| *Specialist literature* | | | | | |
| Group | | | Frequency | Percent | Cumulative Percent |
| 0 Control group | Missing | System | 26 | 100,0 |  |
| 1 Affected group | Valid | no | 9 | 34,6 | 34,6 |
|  |  | yes | 17 | 65,4 | 100,0 |
|  |  | Total | 26 | 100,0 |  |
|  |  |  |  |  |  |
| *Education* | | | | | |
| Group | | | Frequency | Percent | Cumulative Percent |
| 0 Control group | Missing | System | 26 | 100,0 |  |
| 1 Affected group | Valid | no | 22 | 84,6 | 84,6 |
|  |  | yes | 4 | 15,4 | 100,0 |
|  |  | Total | 26 | 100,0 |  |
|  |  |  |  |  |  |
| *Pastoral care* | | | | | |
| Group | | | Frequency | Percent | Cumulative Percent |
| 0 Control group | Missing | System | 26 | 100,0 |  |
| 1 Affected group | Valid | no | 24 | 92,3 | 92,3 |
|  |  | yes | 2 | 7,7 | 100,0 |
|  |  | Total | 26 | 100,0 |  |
|  |  |  |  |  |  |
| *Activity* | | | | | |
| Group | | | Frequency | Percent | Cumulative Percent |
| 0 Control group | Missing | System | 26 | 100,0 |  |
| 1 Affected group | Valid | no | 4 | 15,4 | 15,4 |
|  |  | yes | 22 | 84,6 | 100,0 |
|  |  | Total | 26 | 100,0 |  |
|  |  |  |  |  |  |
| *Psychotherapy* | | | | | |
| Group | | | Frequency | Percent | Cumulative Percent |
| 0 Control group | Missing | System | 26 | 100,0 |  |
| 1 Affected group | Valid | no | 25 | 96,2 | 96,2 |
|  |  | yes | 1 | 3,8 | 100,0 |
|  |  | Total | 26 | 100,0 |  |
|  |  |  |  |  |  |
| *Family subsistence* | | | | | |
| Group | | | Frequency | Percent | Cumulative Percent |
| 0 Control group | Missing | System | 26 | 100,0 |  |
| 1 Affected group | Valid | no | 14 | 53,8 | 53,8 |
|  |  | yes | 12 | 46,2 | 100,0 |
|  |  | Total | 26 | 100,0 |  |
|  |  |  |  |  |  |
| *Parent groups* | | | | | |
| Group | | | Frequency | Percent | Cumulative Percent |
| 0 Control group | Missing | System | 26 | 100,0 |  |
| 1 Affected group | Valid | no | 20 | 76,9 | 76,9 |
|  |  | yes | 6 | 23,1 | 100,0 |
|  |  | Total | 26 | 100,0 |  |
|  |  |  |  |  |  |
| *Being alone* | | | | | |
| Group | | | Frequency | Percent | Cumulative Percent |
| 0 Control group | Missing | System | 26 | 100,0 |  |
| 1 Affected group | Valid | no | 18 | 69,2 | 69,2 |
|  |  | yes | 8 | 30,8 | 100,0 |
|  |  | Total | 26 | 100,0 |  |
|  |  |  |  |  |  |
| *Accusation of God* | | | | | |
| Group | | | Frequency | Percent | Cumulative Percent |
| 0 Control group | Missing | System | 26 | 100,0 |  |
| 1 Affected group | Valid | no | 22 | 84,6 | 84,6 |
|  |  | yes | 4 | 15,4 | 100,0 |
|  |  | Total | 26 | 100,0 |  |
|  |  |  |  |  |  |
| *What has changed: Everything* | | | | | |
| Group | | | Frequency | Percent | Cumulative Percent |
| 0 Control group | Missing | System | 26 | 100,0 |  |
| 1 Affected group | Valid | no | 8 | 30,8 | 30,8 |
|  |  | yes | 18 | 69,2 | 100,0 |
|  |  | Total | 26 | 100,0 |  |
|  |  |  |  |  |  |
| *Nothing* | | | | | |
| Group | | | Frequency | Percent | Cumulative Percent |
| 0 Control group | Missing | System | 26 | 100,0 |  |
| 1 Affected group | Valid | no | 25 | 96,2 | 96,2 |
|  |  | yes | 1 | 3,8 | 100,0 |
|  |  | Total | 26 | 100,0 |  |
|  |  |  |  |  |  |
| *Job* | | | | | |
| Group | | | Frequency | Percent | Cumulative Percent |
| 0 Control group | Missing | System | 26 | 100,0 |  |
| 1 Affected group | Valid | no | 17 | 65,4 | 65,4 |
|  |  | yes | 9 | 34,6 | 100,0 |
|  |  | Total | 26 | 100,0 |  |
|  |  |  |  |  |  |
| *Friends* | | | | | |
| Group | | | Frequency | Percent | Cumulative Percent |
| 0 Control group | Missing | System | 26 | 100,0 |  |
| 1 Affected group | Valid | no | 15 | 57,7 | 57,7 |
|  |  | yes | 11 | 42,3 | 100,0 |
|  |  | Total | 26 | 100,0 |  |
|  |  |  |  |  |  |
| *Relatives* | | | | | |
| Group | | | Frequency | Percent | Cumulative Percent |
| 0 Control group | Missing | System | 26 | 100,0 |  |
| 1 Affected group | Valid | no | 17 | 65,4 | 65,4 |
|  |  | yes | 9 | 34,6 | 100,0 |
|  |  | Total | 26 | 100,0 |  |
|  |  |  |  |  |  |
| *Personality* | | | | | |
| Group | | | Frequency | Percent | Cumulative Percent |
| 0 Control group | Missing | System | 26 | 100,0 |  |
| 1 Affected group | Valid | no | 17 | 65,4 | 65,4 |
|  |  | yes | 9 | 34,6 | 100,0 |
|  |  | Total | 26 | 100,0 |  |
|  |  |  |  |  |  |
| *Environment* | | | | | |
| Group | | | Frequency | Percent | Cumulative Percent |
| 0 Control group | Missing | System | 26 | 100,0 |  |
| 1 Affected group | Valid | no | 18 | 69,2 | 69,2 |
|  |  | yes | 8 | 30,8 | 100,0 |
|  |  | Total | 26 | 100,0 |  |
|  |  |  |  |  |  |
| *General life satisfaction at present: satisfied* | | | | | |
| Group | | | Frequency | Percent | Cumulative Percent |
| 0 Control group | Missing | System | 26 | 100,0 |  |
| 1 Affected group | Valid | no | 13 | 50,0 | 50,0 |
|  |  | yes | 13 | 50,0 | 100,0 |
|  |  | Total | 26 | 100,0 |  |
|  |  |  |  |  |  |
| *Difficult* | | | | | |
| Group | | | Frequency | Percent | Cumulative Percent |
| 0 Control group | Missing | System | 26 | 100,0 |  |
| 1 Affected group | Valid | no | 15 | 57,7 | 57,7 |
|  |  | yes | 11 | 42,3 | 100,0 |
|  |  | Total | 26 | 100,0 |  |
|  |  |  |  |  |  |
| *Unhappy* | | | | | |
| Group | | | Frequency | Percent | Cumulative Percent |
| 0 Control group | Missing | System | 26 | 100,0 |  |
| 1 Affected group | Valid | no | 24 | 92,3 | 92,3 |
|  |  | yes | 2 | 7,7 | 100,0 |
|  |  | Total | 26 | 100,0 |  |
|  |  |  |  |  |  |
| *Disappointed* | | | | | |
| Group | | | Frequency | Percent | Cumulative Percent |
| 0 Control group | Missing | System | 26 | 100,0 |  |
| 1 Affected group | Valid | no | 22 | 84,6 | 84,6 |
|  |  | yes | 4 | 15,4 | 100,0 |
|  |  | Total | 26 | 100,0 |  |
|  |  |  |  |  |  |
| *Happy* | | | | | |
| Group | | | Frequency | Percent | Cumulative Percent |
| 0 Control group | Missing | System | 26 | 100,0 |  |
| 1 Affected group | Valid | no | 18 | 69,2 | 69,2 |
|  |  | yes | 8 | 30,8 | 100,0 |
|  |  | Total | 26 | 100,0 |  |
|  |  |  |  |  |  |
| *Familiar* | | | | | |
| Group | | | Frequency | Percent | Cumulative Percent |
| 0 Control group | Missing | System | 26 | 100,0 |  |
| 1 Affected group | Valid | no | 14 | 53,8 | 53,8 |
|  |  | yes | 12 | 46,2 | 100,0 |
|  |  | Total | 26 | 100,0 |  |

| *FFCv: consolidated* | | | | | |
| --- | --- | --- | --- | --- | --- |
| Group | | | Frequency | Percent | Cumulative Percent |
| 0 Control group | Missing | System | 26 | 100,0 |  |
| 1 Affected group | Valid | no | 14 | 53,8 | 53,8 |
|  |  | yes | 12 | 46,2 | 100,0 |
|  |  | Total | 26 | 100,0 |  |
|  |  |  |  |  |  |
| *Education level* | | | | | |
| Group | | | Frequency | Percent | Cumulative Percent |
| 0 Control group | Valid | Basic school | 7 | 26,9 | 26,9 |
|  |  | Real school | 9 | 34,6 | 61,5 |
|  |  | High school | 7 | 26,9 | 88,5 |
|  |  | College/university | 3 | 11,5 | 100,0 |
|  |  | Total | 26 | 100,0 |  |
| 1 Affected group | Valid | Basic school | 6 | 23,1 | 23,1 |
|  |  | Real school | 6 | 23,1 | 46,2 |
|  |  | High school | 8 | 30,8 | 76,9 |
|  |  | College/university | 6 | 23,1 | 100,0 |
|  |  | Total | 26 | 100,0 |  |
|  |  |  |  |  |  |
| *FERUS motivation to change* | | | | | |
| Group | | | Frequency | Percent | Cumulative Percent |
| 0 Control group | Valid | 28 | 2 | 7,7 | 7,7 |
|  |  | 32 | 2 | 7,7 | 15,4 |
|  |  | 38 | 1 | 3,8 | 19,2 |
|  |  | 40 | 1 | 3,8 | 23,1 |
|  |  | 41 | 1 | 3,8 | 26,9 |
|  |  | 43 | 1 | 3,8 | 30,8 |
|  |  | 44 | 1 | 3,8 | 34,6 |
|  |  | 45 | 1 | 3,8 | 38,5 |
|  |  | 47 | 1 | 3,8 | 42,3 |
|  |  | 48 | 1 | 3,8 | 46,2 |
|  |  | 49 | 2 | 7,7 | 53,8 |
|  |  | 51 | 1 | 3,8 | 57,7 |
|  |  | 52 | 1 | 3,8 | 61,5 |
|  |  | 53 | 1 | 3,8 | 65,4 |
|  |  | 55 | 2 | 7,7 | 73,1 |
|  |  | 60 | 3 | 11,5 | 84,6 |
|  |  | 61 | 2 | 7,7 | 92,3 |
|  |  | 63 | 1 | 3,8 | 96,2 |
|  |  | 69 | 1 | 3,8 | 100,0 |
|  |  | Total | 26 | 100,0 |  |
| 1 Affected group | Valid | 26 | 1 | 3,8 | 3,8 |
|  |  | 28 | 1 | 3,8 | 7,7 |
|  |  | 38 | 1 | 3,8 | 11,5 |
|  |  | 40 | 1 | 3,8 | 15,4 |
|  |  | 43 | 1 | 3,8 | 19,2 |
|  |  | 45 | 1 | 3,8 | 23,1 |
|  |  | 48 | 2 | 7,7 | 30,8 |
|  |  | 49 | 1 | 3,8 | 34,6 |
|  |  | 52 | 1 | 3,8 | 38,5 |
|  |  | 53 | 2 | 7,7 | 46,2 |
|  |  | 57 | 1 | 3,8 | 50,0 |
|  |  | 60 | 3 | 11,5 | 61,5 |
|  |  | 61 | 4 | 15,4 | 76,9 |
|  |  | 63 | 1 | 3,8 | 80,8 |
|  |  | 64 | 1 | 3,8 | 84,6 |
|  |  | 67 | 2 | 7,7 | 92,3 |
|  |  | 69 | 2 | 7,7 | 100,0 |
|  |  | Total | 26 | 100,0 |  |
|  |  |  |  |  |  |
| *FERUS Coping* | | | | | |
| Group | | | Frequency | Percent | Cumulative Percent |
| 0 Control group | Valid | 27 | 1 | 3,8 | 3,8 |
|  |  | 29 | 4 | 15,4 | 19,2 |
|  |  | 35 | 1 | 3,8 | 23,1 |
|  |  | 37 | 1 | 3,8 | 26,9 |
|  |  | 39 | 1 | 3,8 | 30,8 |
|  |  | 41 | 3 | 11,5 | 42,3 |
|  |  | 43 | 2 | 7,7 | 50,0 |
|  |  | 45 | 2 | 7,7 | 57,7 |
|  |  | 47 | 2 | 7,7 | 65,4 |
|  |  | 48 | 2 | 7,7 | 73,1 |
|  |  | 50 | 2 | 7,7 | 80,8 |
|  |  | 56 | 1 | 3,8 | 84,6 |
|  |  | 62 | 1 | 3,8 | 88,5 |
|  |  | 68 | 3 | 11,5 | 100,0 |
|  |  | Total | 26 | 100,0 |  |
| 1 Affected group | Valid | 26 | 1 | 3,8 | 3,8 |
|  |  | 29 | 1 | 3,8 | 7,7 |
|  |  | 35 | 3 | 11,5 | 19,2 |
|  |  | 42 | 1 | 3,8 | 23,1 |
|  |  | 43 | 2 | 7,7 | 30,8 |
|  |  | 45 | 4 | 15,4 | 46,2 |
|  |  | 47 | 1 | 3,8 | 50,0 |
|  |  | 48 | 1 | 3,8 | 53,8 |
|  |  | 50 | 1 | 3,8 | 57,7 |
|  |  | 52 | 1 | 3,8 | 61,5 |
|  |  | 54 | 1 | 3,8 | 65,4 |
|  |  | 55 | 1 | 3,8 | 69,2 |
|  |  | 56 | 2 | 7,7 | 76,9 |
|  |  | 57 | 1 | 3,8 | 80,8 |
|  |  | 59 | 2 | 7,7 | 88,5 |
|  |  | 60 | 1 | 3,8 | 92,3 |
|  |  | 62 | 1 | 3,8 | 96,2 |
|  |  | 71 | 1 | 3,8 | 100,0 |
|  |  | Total | 26 | 100,0 |  |
|  |  |  |  |  |  |
| *FERUS Self-monitoring* | | | | | |
| Group | | | Frequency | Percent | Cumulative Percent |
| 0 Control group | Valid | 31 | 2 | 7,7 | 7,7 |
|  |  | 34 | 1 | 3,8 | 11,5 |
|  |  | 35 | 1 | 3,8 | 15,4 |
|  |  | 37 | 2 | 7,7 | 23,1 |
|  |  | 39 | 3 | 11,5 | 34,6 |
|  |  | 43 | 1 | 3,8 | 38,5 |
|  |  | 44 | 1 | 3,8 | 42,3 |
|  |  | 45 | 1 | 3,8 | 46,2 |
|  |  | 46 | 2 | 7,7 | 53,8 |
|  |  | 49 | 1 | 3,8 | 57,7 |
|  |  | 51 | 1 | 3,8 | 61,5 |
|  |  | 54 | 4 | 15,4 | 76,9 |
|  |  | 57 | 1 | 3,8 | 80,8 |
|  |  | 63 | 3 | 11,5 | 92,3 |
|  |  | 64 | 1 | 3,8 | 96,2 |
|  |  | 66 | 1 | 3,8 | 100,0 |
|  |  | Total | 26 | 100,0 |  |
| 1 Affected group | Valid | 34 | 1 | 3,8 | 3,8 |
|  |  | 35 | 1 | 3,8 | 7,7 |
|  |  | 36 | 3 | 11,5 | 19,2 |
|  |  | 39 | 2 | 7,7 | 26,9 |
|  |  | 41 | 4 | 15,4 | 42,3 |
|  |  | 43 | 1 | 3,8 | 46,2 |
|  |  | 44 | 1 | 3,8 | 50,0 |
|  |  | 46 | 3 | 11,5 | 61,5 |
|  |  | 49 | 2 | 7,7 | 69,2 |
|  |  | 51 | 2 | 7,7 | 76,9 |
|  |  | 54 | 2 | 7,7 | 84,6 |
|  |  | 57 | 2 | 7,7 | 92,3 |
|  |  | 59 | 2 | 7,7 | 100,0 |
|  |  | Total | 26 | 100,0 |  |
|  |  |  |  |  |  |
| *FERUS Self-efficacy* | | | | | |
| Group | | | Frequency | Percent | Cumulative Percent |
| 0 Control group | Valid | 26 | 1 | 3,8 | 3,8 |
|  |  | 31 | 2 | 7,7 | 11,5 |
|  |  | 35 | 2 | 7,7 | 19,2 |
|  |  | 37 | 4 | 15,4 | 34,6 |
|  |  | 38 | 1 | 3,8 | 38,5 |
|  |  | 39 | 2 | 7,7 | 46,2 |
|  |  | 40 | 1 | 3,8 | 50,0 |
|  |  | 43 | 3 | 11,5 | 61,5 |
|  |  | 46 | 1 | 3,8 | 65,4 |
|  |  | 48 | 2 | 7,7 | 73,1 |
|  |  | 52 | 3 | 11,5 | 84,6 |
|  |  | 54 | 4 | 15,4 | 100,0 |
|  |  | Total | 26 | 100,0 |  |
| 1 Affected group | Valid | 26 | 1 | 3,8 | 3,8 |
|  |  | 31 | 2 | 7,7 | 11,5 |
|  |  | 37 | 2 | 7,7 | 19,2 |
|  |  | 39 | 1 | 3,8 | 23,1 |
|  |  | 40 | 1 | 3,8 | 26,9 |
|  |  | 43 | 1 | 3,8 | 30,8 |
|  |  | 45 | 4 | 15,4 | 46,2 |
|  |  | 48 | 1 | 3,8 | 50,0 |
|  |  | 50 | 2 | 7,7 | 57,7 |
|  |  | 52 | 3 | 11,5 | 69,2 |
|  |  | 54 | 4 | 15,4 | 84,6 |
|  |  | 57 | 3 | 11,5 | 96,2 |
|  |  | 58 | 1 | 3,8 | 100,0 |
|  |  | Total | 26 | 100,0 |  |
|  |  |  |  |  |  |
| *FERUS Self-verbalization* | | | | | |
| Group | | | Frequency | Percent | Cumulative Percent |
| 0 Control group | Valid | 22 | 1 | 3,8 | 3,8 |
|  |  | 25 | 1 | 3,8 | 7,7 |
|  |  | 26 | 1 | 3,8 | 11,5 |
|  |  | 29 | 1 | 3,8 | 15,4 |
|  |  | 34 | 1 | 3,8 | 19,2 |
|  |  | 39 | 1 | 3,8 | 23,1 |
|  |  | 41 | 2 | 7,7 | 30,8 |
|  |  | 42 | 3 | 11,5 | 42,3 |
|  |  | 43 | 2 | 7,7 | 50,0 |
|  |  | 44 | 2 | 7,7 | 57,7 |
|  |  | 46 | 3 | 11,5 | 69,2 |
|  |  | 52 | 3 | 11,5 | 80,8 |
|  |  | 59 | 2 | 7,7 | 88,5 |
|  |  | 61 | 2 | 7,7 | 96,2 |
|  |  | 72 | 1 | 3,8 | 100,0 |
|  |  | Total | 26 | 100,0 |  |
| 1 Affected group | Valid | 26 | 1 | 3,8 | 3,8 |
|  |  | 34 | 1 | 3,8 | 7,7 |
|  |  | 38 | 1 | 3,8 | 11,5 |
|  |  | 39 | 1 | 3,8 | 15,4 |
|  |  | 41 | 1 | 3,8 | 19,2 |
|  |  | 43 | 2 | 7,7 | 26,9 |
|  |  | 44 | 3 | 11,5 | 38,5 |
|  |  | 46 | 2 | 7,7 | 46,2 |
|  |  | 49 | 2 | 7,7 | 53,8 |
|  |  | 52 | 3 | 11,5 | 65,4 |
|  |  | 55 | 3 | 11,5 | 76,9 |
|  |  | 57 | 3 | 11,5 | 88,5 |
|  |  | 59 | 2 | 7,7 | 96,2 |
|  |  | 72 | 1 | 3,8 | 100,0 |
|  |  | Total | 26 | 100,0 |  |
|  |  |  |  |  |  |
| *FERUS Hope* | | | | | |
| Group | | | Frequency | Percent | Cumulative Percent |
| 0 Control group | Valid | 26 | 1 | 3,8 | 3,8 |
|  |  | 30 | 1 | 3,8 | 7,7 |
|  |  | 33 | 3 | 11,5 | 19,2 |
|  |  | 36 | 2 | 7,7 | 26,9 |
|  |  | 39 | 1 | 3,8 | 30,8 |
|  |  | 40 | 1 | 3,8 | 34,6 |
|  |  | 41 | 2 | 7,7 | 42,3 |
|  |  | 42 | 1 | 3,8 | 46,2 |
|  |  | 43 | 5 | 19,2 | 65,4 |
|  |  | 44 | 1 | 3,8 | 69,2 |
|  |  | 46 | 2 | 7,7 | 76,9 |
|  |  | 48 | 1 | 3,8 | 80,8 |
|  |  | 51 | 2 | 7,7 | 88,5 |
|  |  | 55 | 2 | 7,7 | 96,2 |
|  |  | 57 | 1 | 3,8 | 100,0 |
|  |  | Total | 26 | 100,0 |  |
| 1 Affected group | Valid | 26 | 3 | 11,5 | 11,5 |
|  |  | 33 | 3 | 11,5 | 23,1 |
|  |  | 36 | 1 | 3,8 | 26,9 |
|  |  | 39 | 1 | 3,8 | 30,8 |
|  |  | 40 | 1 | 3,8 | 34,6 |
|  |  | 41 | 2 | 7,7 | 42,3 |
|  |  | 42 | 1 | 3,8 | 46,2 |
|  |  | 43 | 1 | 3,8 | 50,0 |
|  |  | 45 | 1 | 3,8 | 53,8 |
|  |  | 49 | 6 | 23,1 | 76,9 |
|  |  | 51 | 1 | 3,8 | 80,8 |
|  |  | 55 | 3 | 11,5 | 92,3 |
|  |  | 61 | 1 | 3,8 | 96,2 |
|  |  | 63 | 1 | 3,8 | 100,0 |
|  |  | Total | 26 | 100,0 |  |
|  |  |  |  |  |  |
| *FERUS Social support* | | | | | |
| Group | | | Frequency | Percent | Cumulative Percent |
| 0 Control group | Valid | 26 | 1 | 3,8 | 3,8 |
|  |  | 28 | 1 | 3,8 | 7,7 |
|  |  | 30 | 1 | 3,8 | 11,5 |
|  |  | 33 | 4 | 15,4 | 26,9 |
|  |  | 34 | 2 | 7,7 | 34,6 |
|  |  | 38 | 2 | 7,7 | 42,3 |
|  |  | 39 | 2 | 7,7 | 50,0 |
|  |  | 40 | 3 | 11,5 | 61,5 |
|  |  | 44 | 1 | 3,8 | 65,4 |
|  |  | 46 | 1 | 3,8 | 69,2 |
|  |  | 47 | 1 | 3,8 | 73,1 |
|  |  | 48 | 5 | 19,2 | 92,3 |
|  |  | 50 | 2 | 7,7 | 100,0 |
|  |  | Total | 26 | 100,0 |  |
| 1 Affected group | Valid | 26 | 1 | 3,8 | 3,8 |
|  |  | 33 | 2 | 7,7 | 11,5 |
|  |  | 34 | 3 | 11,5 | 23,1 |
|  |  | 36 | 1 | 3,8 | 26,9 |
|  |  | 38 | 1 | 3,8 | 30,8 |
|  |  | 39 | 2 | 7,7 | 38,5 |
|  |  | 40 | 4 | 15,4 | 53,8 |
|  |  | 42 | 2 | 7,7 | 61,5 |
|  |  | 44 | 2 | 7,7 | 69,2 |
|  |  | 46 | 2 | 7,7 | 76,9 |
|  |  | 48 | 1 | 3,8 | 80,8 |
|  |  | 49 | 1 | 3,8 | 84,6 |
|  |  | 50 | 2 | 7,7 | 92,3 |
|  |  | 57 | 2 | 7,7 | 100,0 |
|  |  | Total | 26 | 100,0 |  |
|  |  |  |  |  |  |
| *FERUS total Self-management skills* | | | | | |
| Group | | | Frequency | Percent | Cumulative Percent |
| 0 Control group | Valid | 26 | 1 | 3,8 | 3,8 |
|  |  | 36 | 1 | 3,8 | 7,7 |
|  |  | 37 | 2 | 7,7 | 15,4 |
|  |  | 39 | 2 | 7,7 | 23,1 |
|  |  | 41 | 2 | 7,7 | 30,8 |
|  |  | 42 | 1 | 3,8 | 34,6 |
|  |  | 45 | 2 | 7,7 | 42,3 |
|  |  | 46 | 1 | 3,8 | 46,2 |
|  |  | 47 | 1 | 3,8 | 50,0 |
|  |  | 48 | 3 | 11,5 | 61,5 |
|  |  | 50 | 1 | 3,8 | 65,4 |
|  |  | 55 | 2 | 7,7 | 73,1 |
|  |  | 58 | 1 | 3,8 | 76,9 |
|  |  | 59 | 1 | 3,8 | 80,8 |
|  |  | 62 | 1 | 3,8 | 84,6 |
|  |  | 71 | 1 | 3,8 | 88,5 |
|  |  | 74 | 3 | 11,5 | 100,0 |
|  |  | Total | 26 | 100,0 |  |
| 1 Affected group | Valid | 26 | 2 | 7,7 | 7,7 |
|  |  | 32 | 1 | 3,8 | 11,5 |
|  |  | 34 | 1 | 3,8 | 15,4 |
|  |  | 36 | 5 | 19,2 | 34,6 |
|  |  | 37 | 3 | 11,5 | 46,2 |
|  |  | 40 | 1 | 3,8 | 50,0 |
|  |  | 43 | 1 | 3,8 | 53,8 |
|  |  | 45 | 3 | 11,5 | 65,4 |
|  |  | 46 | 3 | 11,5 | 76,9 |
|  |  | 49 | 1 | 3,8 | 80,8 |
|  |  | 50 | 3 | 11,5 | 92,3 |
|  |  | 52 | 1 | 3,8 | 96,2 |
|  |  | 55 | 1 | 3,8 | 100,0 |
|  |  | Total | 26 | 100,0 |  |
|  |  |  |  |  |  |
| *Religion* | | | | | |
| Group | | | Frequency | Percent | Cumulative Percent |
| 0 Control group | Valid | none | 1 | 3,8 | 3,8 |
|  |  | Roman Catholic | 20 | 76,9 | 80,8 |
|  |  | Protestant | 5 | 19,2 | 100,0 |
|  |  | Total | 26 | 100,0 |  |
| 1 Affected group | Valid | none | 6 | 23,1 | 23,1 |
|  |  | Roman Catholic | 7 | 26,9 | 50,0 |
|  |  | Protestant | 12 | 46,2 | 96,2 |
|  |  | Other | 1 | 3,8 | 100,0 |
|  |  | Total | 26 | 100,0 |  |
|  |  |  |  |  |  |
| *SEE Acceptance of emotions* | | | | | |
| Group | | | Frequency | Percent | Cumulative Percent |
| 0 Control group | Valid | 22 | 2 | 7,7 | 7,7 |
|  |  | 23 | 1 | 3,8 | 11,5 |
|  |  | 24 | 1 | 3,8 | 15,4 |
|  |  | 33 | 1 | 3,8 | 19,2 |
|  |  | 39 | 2 | 7,7 | 26,9 |
|  |  | 41 | 1 | 3,8 | 30,8 |
|  |  | 42 | 1 | 3,8 | 34,6 |
|  |  | 44 | 1 | 3,8 | 38,5 |
|  |  | 46 | 1 | 3,8 | 42,3 |
|  |  | 48 | 2 | 7,7 | 50,0 |
|  |  | 51 | 1 | 3,8 | 53,8 |
|  |  | 53 | 1 | 3,8 | 57,7 |
|  |  | 55 | 2 | 7,7 | 65,4 |
|  |  | 57 | 3 | 11,5 | 76,9 |
|  |  | 59 | 2 | 7,7 | 84,6 |
|  |  | 61 | 1 | 3,8 | 88,5 |
|  |  | 64 | 2 | 7,7 | 96,2 |
|  |  | 68 | 1 | 3,8 | 100,0 |
|  |  | Total | 26 | 100,0 |  |
| 1 Affected group | Valid | 24 | 1 | 3,8 | 3,8 |
|  |  | 28 | 1 | 3,8 | 7,7 |
|  |  | 39 | 1 | 3,8 | 11,5 |
|  |  | 44 | 4 | 15,4 | 26,9 |
|  |  | 46 | 1 | 3,8 | 30,8 |
|  |  | 48 | 4 | 15,4 | 46,2 |
|  |  | 50 | 2 | 7,7 | 53,8 |
|  |  | 52 | 1 | 3,8 | 57,7 |
|  |  | 53 | 5 | 19,2 | 76,9 |
|  |  | 55 | 2 | 7,7 | 84,6 |
|  |  | 64 | 3 | 11,5 | 96,2 |
|  |  | 66 | 1 | 3,8 | 100,0 |
|  |  | Total | 26 | 100,0 |  |
|  |  |  |  |  |  |
| *SEE Emotion regulation* | | | | | |
| Group | | | Frequency | Percent | Cumulative Percent |
| 0 Control group | Valid | 13 | 1 | 3,8 | 3,8 |
|  |  | 28 | 2 | 7,7 | 11,5 |
|  |  | 31 | 1 | 3,8 | 15,4 |
|  |  | 39 | 2 | 7,7 | 23,1 |
|  |  | 43 | 3 | 11,5 | 34,6 |
|  |  | 46 | 3 | 11,5 | 46,2 |
|  |  | 49 | 1 | 3,8 | 50,0 |
|  |  | 50 | 1 | 3,8 | 53,8 |
|  |  | 52 | 1 | 3,8 | 57,7 |
|  |  | 54 | 1 | 3,8 | 61,5 |
|  |  | 55 | 2 | 7,7 | 69,2 |
|  |  | 56 | 1 | 3,8 | 73,1 |
|  |  | 57 | 2 | 7,7 | 80,8 |
|  |  | 61 | 2 | 7,7 | 88,5 |
|  |  | 65 | 1 | 3,8 | 92,3 |
|  |  | 69 | 1 | 3,8 | 96,2 |
|  |  | 80 | 1 | 3,8 | 100,0 |
|  |  | Total | 26 | 100,0 |  |
| 1 Affected group | Valid | 28 | 2 | 7,7 | 7,7 |
|  |  | 31 | 2 | 7,7 | 15,4 |
|  |  | 43 | 2 | 7,7 | 23,1 |
|  |  | 46 | 5 | 19,2 | 42,3 |
|  |  | 50 | 3 | 11,5 | 53,8 |
|  |  | 54 | 4 | 15,4 | 69,2 |
|  |  | 57 | 2 | 7,7 | 76,9 |
|  |  | 59 | 1 | 3,8 | 80,8 |
|  |  | 61 | 4 | 15,4 | 96,2 |
|  |  | 65 | 1 | 3,8 | 100,0 |
|  |  | Total | 26 | 100,0 |  |
|  |  |  |  |  |  |
| *SEE: Emotion overflow* | | | | | |
| Group | | | Frequency | Percent | Cumulative Percent |
| 0 Control group | Valid | 17 | 1 | 3,8 | 3,8 |
|  |  | 38 | 1 | 3,8 | 7,7 |
|  |  | 43 | 2 | 7,7 | 15,4 |
|  |  | 45 | 3 | 11,5 | 26,9 |
|  |  | 49 | 1 | 3,8 | 30,8 |
|  |  | 50 | 3 | 11,5 | 42,3 |
|  |  | 52 | 2 | 7,7 | 50,0 |
|  |  | 54 | 2 | 7,7 | 57,7 |
|  |  | 55 | 2 | 7,7 | 65,4 |
|  |  | 56 | 1 | 3,8 | 69,2 |
|  |  | 57 | 1 | 3,8 | 73,1 |
|  |  | 59 | 3 | 11,5 | 84,6 |
|  |  | 64 | 2 | 7,7 | 92,3 |
|  |  | 65 | 1 | 3,8 | 96,2 |
|  |  | 66 | 1 | 3,8 | 100,0 |
|  |  | Total | 26 | 100,0 |  |
| 1 Affected group | Valid | 33 | 1 | 3,8 | 3,8 |
|  |  | 35 | 1 | 3,8 | 7,7 |
|  |  | 38 | 1 | 3,8 | 11,5 |
|  |  | 40 | 1 | 3,8 | 15,4 |
|  |  | 41 | 1 | 3,8 | 19,2 |
|  |  | 42 | 1 | 3,8 | 23,1 |
|  |  | 43 | 2 | 7,7 | 30,8 |
|  |  | 45 | 1 | 3,8 | 34,6 |
|  |  | 47 | 4 | 15,4 | 50,0 |
|  |  | 49 | 1 | 3,8 | 53,8 |
|  |  | 50 | 1 | 3,8 | 57,7 |
|  |  | 52 | 3 | 11,5 | 69,2 |
|  |  | 54 | 3 | 11,5 | 80,8 |
|  |  | 55 | 1 | 3,8 | 84,6 |
|  |  | 59 | 1 | 3,8 | 88,5 |
|  |  | 61 | 1 | 3,8 | 92,3 |
|  |  | 64 | 1 | 3,8 | 96,2 |
|  |  | 73 | 1 | 3,8 | 100,0 |
|  |  | Total | 26 | 100,0 |  |
|  |  |  |  |  |  |
| *SEE: Control* | | | | | |
| Group | | | Frequency | Percent | Cumulative Percent |
| 0 Control group | Valid | 14 | 1 | 3,8 | 3,8 |
|  |  | 30 | 2 | 7,7 | 11,5 |
|  |  | 35 | 1 | 3,8 | 15,4 |
|  |  | 37 | 2 | 7,7 | 23,1 |
|  |  | 40 | 2 | 7,7 | 30,8 |
|  |  | 43 | 1 | 3,8 | 34,6 |
|  |  | 44 | 1 | 3,8 | 38,5 |
|  |  | 47 | 2 | 7,7 | 46,2 |
|  |  | 49 | 4 | 15,4 | 61,5 |
|  |  | 51 | 2 | 7,7 | 69,2 |
|  |  | 53 | 1 | 3,8 | 73,1 |
|  |  | 56 | 1 | 3,8 | 76,9 |
|  |  | 60 | 2 | 7,7 | 84,6 |
|  |  | 67 | 1 | 3,8 | 88,5 |
|  |  | 71 | 2 | 7,7 | 96,2 |
|  |  | 74 | 1 | 3,8 | 100,0 |
|  |  | Total | 26 | 100,0 |  |
| 1 Affected group | Valid | 35 | 1 | 3,8 | 3,8 |
|  |  | 37 | 1 | 3,8 | 7,7 |
|  |  | 40 | 1 | 3,8 | 11,5 |
|  |  | 49 | 4 | 15,4 | 26,9 |
|  |  | 51 | 1 | 3,8 | 30,8 |
|  |  | 53 | 2 | 7,7 | 38,5 |
|  |  | 56 | 3 | 11,5 | 50,0 |
|  |  | 57 | 1 | 3,8 | 53,8 |
|  |  | 58 | 3 | 11,5 | 65,4 |
|  |  | 60 | 2 | 7,7 | 73,1 |
|  |  | 62 | 4 | 15,4 | 88,5 |
|  |  | 65 | 1 | 3,8 | 92,3 |
|  |  | 67 | 2 | 7,7 | 100,0 |
|  |  | Total | 26 | 100,0 |  |
|  |  |  |  |  |  |
| *SEE: Loss of emotions* | | | | | |
| Group | | | Frequency | Percent | Cumulative Percent |
| 0 Control group | Valid | 11 | 1 | 3,8 | 3,8 |
|  |  | 36 | 1 | 3,8 | 7,7 |
|  |  | 39 | 3 | 11,5 | 19,2 |
|  |  | 41 | 3 | 11,5 | 30,8 |
|  |  | 44 | 1 | 3,8 | 34,6 |
|  |  | 46 | 1 | 3,8 | 38,5 |
|  |  | 48 | 1 | 3,8 | 42,3 |
|  |  | 50 | 2 | 7,7 | 50,0 |
|  |  | 52 | 3 | 11,5 | 61,5 |
|  |  | 57 | 1 | 3,8 | 65,4 |
|  |  | 61 | 1 | 3,8 | 69,2 |
|  |  | 63 | 4 | 15,4 | 84,6 |
|  |  | 64 | 1 | 3,8 | 88,5 |
|  |  | 66 | 3 | 11,5 | 100,0 |
|  |  | Total | 26 | 100,0 |  |
| 1 Affected group | Valid | 31 | 1 | 3,8 | 3,8 |
|  |  | 33 | 1 | 3,8 | 7,7 |
|  |  | 36 | 3 | 11,5 | 19,2 |
|  |  | 37 | 1 | 3,8 | 23,1 |
|  |  | 39 | 1 | 3,8 | 26,9 |
|  |  | 44 | 3 | 11,5 | 38,5 |
|  |  | 47 | 3 | 11,5 | 50,0 |
|  |  | 50 | 2 | 7,7 | 57,7 |
|  |  | 52 | 4 | 15,4 | 73,1 |
|  |  | 58 | 2 | 7,7 | 80,8 |
|  |  | 61 | 1 | 3,8 | 84,6 |
|  |  | 63 | 1 | 3,8 | 88,5 |
|  |  | 72 | 1 | 3,8 | 92,3 |
|  |  | 74 | 1 | 3,8 | 96,2 |
|  |  | 77 | 1 | 3,8 | 100,0 |
|  |  | Total | 26 | 100,0 |  |
|  |  |  |  |  |  |
| *SEE: Bodily expression of emotions* | | | | | |
| Group | | | Frequency | Percent | Cumulative Percent |
| 0 Control group | Valid | 30 | 1 | 3,8 | 3,8 |
|  |  | 32 | 1 | 3,8 | 7,7 |
|  |  | 35 | 1 | 3,8 | 11,5 |
|  |  | 39 | 2 | 7,7 | 19,2 |
|  |  | 41 | 3 | 11,5 | 30,8 |
|  |  | 43 | 1 | 3,8 | 34,6 |
|  |  | 44 | 1 | 3,8 | 38,5 |
|  |  | 47 | 1 | 3,8 | 42,3 |
|  |  | 49 | 3 | 11,5 | 53,8 |
|  |  | 51 | 1 | 3,8 | 57,7 |
|  |  | 52 | 3 | 11,5 | 69,2 |
|  |  | 53 | 2 | 7,7 | 76,9 |
|  |  | 54 | 1 | 3,8 | 80,8 |
|  |  | 55 | 1 | 3,8 | 84,6 |
|  |  | 58 | 1 | 3,8 | 88,5 |
|  |  | 61 | 3 | 11,5 | 100,0 |
|  |  | Total | 26 | 100,0 |  |
| 1 Affected group | Valid | 32 | 1 | 3,8 | 3,8 |
|  |  | 34 | 1 | 3,8 | 7,7 |
|  |  | 39 | 1 | 3,8 | 11,5 |
|  |  | 41 | 5 | 19,2 | 30,8 |
|  |  | 43 | 2 | 7,7 | 38,5 |
|  |  | 45 | 1 | 3,8 | 42,3 |
|  |  | 48 | 1 | 3,8 | 46,2 |
|  |  | 49 | 1 | 3,8 | 50,0 |
|  |  | 51 | 3 | 11,5 | 61,5 |
|  |  | 52 | 3 | 11,5 | 73,1 |
|  |  | 54 | 2 | 7,7 | 80,8 |
|  |  | 56 | 2 | 7,7 | 88,5 |
|  |  | 58 | 1 | 3,8 | 92,3 |
|  |  | 60 | 2 | 7,7 | 100,0 |
|  |  | Total | 26 | 100,0 |  |
|  |  |  |  |  |  |
| *SEE: Imaginative symbolization of emotions* | | | | | |
| Group | | | Frequency | Percent | Cumulative Percent |
| 0 Control group | Valid | 20 | 1 | 3,8 | 3,8 |
|  |  | 27 | 1 | 3,8 | 7,7 |
|  |  | 33 | 2 | 7,7 | 15,4 |
|  |  | 37 | 1 | 3,8 | 19,2 |
|  |  | 41 | 3 | 11,5 | 30,8 |
|  |  | 46 | 2 | 7,7 | 38,5 |
|  |  | 48 | 3 | 11,5 | 50,0 |
|  |  | 49 | 2 | 7,7 | 57,7 |
|  |  | 54 | 1 | 3,8 | 61,5 |
|  |  | 56 | 1 | 3,8 | 65,4 |
|  |  | 57 | 1 | 3,8 | 69,2 |
|  |  | 59 | 2 | 7,7 | 76,9 |
|  |  | 61 | 3 | 11,5 | 88,5 |
|  |  | 72 | 3 | 11,5 | 100,0 |
|  |  | Total | 26 | 100,0 |  |
| 1 Affected group | Valid | 33 | 1 | 3,8 | 3,8 |
|  |  | 35 | 1 | 3,8 | 7,7 |
|  |  | 38 | 1 | 3,8 | 11,5 |
|  |  | 41 | 3 | 11,5 | 23,1 |
|  |  | 42 | 2 | 7,7 | 30,8 |
|  |  | 44 | 2 | 7,7 | 38,5 |
|  |  | 48 | 1 | 3,8 | 42,3 |
|  |  | 50 | 4 | 15,4 | 57,7 |
|  |  | 56 | 2 | 7,7 | 65,4 |
|  |  | 57 | 2 | 7,7 | 73,1 |
|  |  | 59 | 3 | 11,5 | 84,6 |
|  |  | 63 | 1 | 3,8 | 88,5 |
|  |  | 65 | 1 | 3,8 | 92,3 |
|  |  | 67 | 2 | 7,7 | 100,0 |
|  |  | Total | 26 | 100,0 |  |
|  |  |  |  |  |  |
| *SEE total raw value* | | | | | |
| Group | | | Frequency | Percent | Cumulative Percent |
| 0 Control group | Valid | 105 | 2 | 7,7 | 7,7 |
|  |  | 108 | 1 | 3,8 | 11,5 |
|  |  | 112 | 1 | 3,8 | 15,4 |
|  |  | 114 | 1 | 3,8 | 19,2 |
|  |  | 117 | 1 | 3,8 | 23,1 |
|  |  | 120 | 1 | 3,8 | 26,9 |
|  |  | 122 | 1 | 3,8 | 30,8 |
|  |  | 123 | 2 | 7,7 | 38,5 |
|  |  | 125 | 2 | 7,7 | 46,2 |
|  |  | 127 | 1 | 3,8 | 50,0 |
|  |  | 128 | 1 | 3,8 | 53,8 |
|  |  | 129 | 1 | 3,8 | 57,7 |
|  |  | 134 | 1 | 3,8 | 61,5 |
|  |  | 136 | 4 | 15,4 | 76,9 |
|  |  | 138 | 1 | 3,8 | 80,8 |
|  |  | 139 | 1 | 3,8 | 84,6 |
|  |  | 146 | 1 | 3,8 | 88,5 |
|  |  | 153 | 1 | 3,8 | 92,3 |
|  |  | 156 | 2 | 7,7 | 100,0 |
|  |  | Total | 26 | 100,0 |  |
| 1 Affected group | Valid | 107 | 1 | 3,8 | 3,8 |
|  |  | 108 | 1 | 3,8 | 7,7 |
|  |  | 109 | 3 | 11,5 | 19,2 |
|  |  | 112 | 1 | 3,8 | 23,1 |
|  |  | 116 | 1 | 3,8 | 26,9 |
|  |  | 117 | 1 | 3,8 | 30,8 |
|  |  | 118 | 1 | 3,8 | 34,6 |
|  |  | 119 | 2 | 7,7 | 42,3 |
|  |  | 126 | 2 | 7,7 | 50,0 |
|  |  | 128 | 1 | 3,8 | 53,8 |
|  |  | 130 | 1 | 3,8 | 57,7 |
|  |  | 131 | 1 | 3,8 | 61,5 |
|  |  | 132 | 1 | 3,8 | 65,4 |
|  |  | 135 | 1 | 3,8 | 69,2 |
|  |  | 136 | 3 | 11,5 | 80,8 |
|  |  | 137 | 1 | 3,8 | 84,6 |
|  |  | 138 | 2 | 7,7 | 92,3 |
|  |  | 150 | 1 | 3,8 | 96,2 |
|  |  | 159 | 1 | 3,8 | 100,0 |
|  |  | Total | 26 | 100,0 |  |
|  |  |  |  |  |  |
| *FLZ health (stanine value)* | | | | | |
| Group | | | Frequency | Percent | Cumulative Percent |
| 0 Control group | Valid | 1 | 5 | 19,2 | 19,2 |
|  |  | 2 | 5 | 19,2 | 38,5 |
|  |  | 3 | 5 | 19,2 | 57,7 |
|  |  | 4 | 2 | 7,7 | 65,4 |
|  |  | 5 | 4 | 15,4 | 80,8 |
|  |  | 6 | 3 | 11,5 | 92,3 |
|  |  | 7 | 1 | 3,8 | 96,2 |
|  |  | 8 | 1 | 3,8 | 100,0 |
|  |  | Total | 26 | 100,0 |  |
| 1 Affected group | Valid | 1 | 1 | 3,8 | 3,8 |
|  |  | 2 | 6 | 23,1 | 26,9 |
|  |  | 3 | 6 | 23,1 | 50,0 |
|  |  | 4 | 1 | 3,8 | 53,8 |
|  |  | 5 | 6 | 23,1 | 76,9 |
|  |  | 6 | 3 | 11,5 | 88,5 |
|  |  | 7 | 1 | 3,8 | 92,3 |
|  |  | 8 | 2 | 7,7 | 100,0 |
|  |  | Total | 26 | 100,0 |  |
|  |  |  |  |  |  |
| *FLZ job and occupation (stanine value)* | | | | | |
| Group | | | Frequency | Percent | Cumulative Percent |
| 0 Control group | Valid | 1 | 1 | 3,8 | 3,8 |
|  |  | 3 | 1 | 3,8 | 7,7 |
|  |  | 4 | 10 | 38,5 | 46,2 |
|  |  | 5 | 5 | 19,2 | 65,4 |
|  |  | 6 | 7 | 26,9 | 92,3 |
|  |  | 8 | 2 | 7,7 | 100,0 |
|  |  | Total | 26 | 100,0 |  |
| 1 Affected group | Valid | 1 | 2 | 7,7 | 7,7 |
|  |  | 2 | 1 | 3,8 | 11,5 |
|  |  | 3 | 4 | 15,4 | 26,9 |
|  |  | 4 | 2 | 7,7 | 34,6 |
|  |  | 5 | 5 | 19,2 | 53,8 |
|  |  | 7 | 6 | 23,1 | 76,9 |
|  |  | 8 | 2 | 7,7 | 84,6 |
|  |  | Missing | 4 | 15,4 | 100,0 |
|  |  | Total | 26 | 100,0 |  |
|  |  |  |  |  |  |
| *FLZ financial situation (stanine value)* | | | | | |
| Group | | | Frequency | Percent | Cumulative Percent |
| 0 Control group | Valid | 1 | 1 | 3,8 | 3,8 |
|  |  | 3 | 4 | 15,4 | 19,2 |
|  |  | 4 | 7 | 26,9 | 46,2 |
|  |  | 5 | 1 | 3,8 | 50,0 |
|  |  | 6 | 7 | 26,9 | 76,9 |
|  |  | 7 | 4 | 15,4 | 92,3 |
|  |  | 8 | 1 | 3,8 | 96,2 |
|  |  | 9 | 1 | 3,8 | 100,0 |
|  |  | Total | 26 | 100,0 |  |
| 1 Affected group | Valid | 2 | 4 | 15,4 | 15,4 |
|  |  | 3 | 3 | 11,5 | 26,9 |
|  |  | 4 | 3 | 11,5 | 38,5 |
|  |  | 5 | 8 | 30,8 | 69,2 |
|  |  | 6 | 1 | 3,8 | 73,1 |
|  |  | 7 | 4 | 15,4 | 88,5 |
|  |  | Missing | 3 | 11,5 | 100,0 |
|  |  | Total | 26 | 100,0 |  |
|  |  |  |  |  |  |
| *FLZ leisure (stanine value)* | | | | | |
| Group | | | Frequency | Percent | Cumulative Percent |
| 0 Control group | Valid | 2 | 1 | 3,8 | 3,8 |
|  |  | 3 | 4 | 15,4 | 19,2 |
|  |  | 4 | 8 | 30,8 | 50,0 |
|  |  | 5 | 5 | 19,2 | 69,2 |
|  |  | 6 | 6 | 23,1 | 92,3 |
|  |  | 7 | 2 | 7,7 | 100,0 |
|  |  | Total | 26 | 100,0 |  |
| 1 Affected group | Valid | 1 | 7 | 26,9 | 26,9 |
|  |  | 2 | 6 | 23,1 | 50,0 |
|  |  | 3 | 2 | 7,7 | 57,7 |
|  |  | 4 | 3 | 11,5 | 69,2 |
|  |  | 5 | 3 | 11,5 | 80,8 |
|  |  | 8 | 1 | 3,8 | 84,6 |
|  |  | Missing | 4 | 15,4 | 100,0 |
|  |  | Total | 26 | 100,0 |  |
|  |  |  |  |  |  |
| *FLZ partnership (stanine value)* | | | | | |
| Group | | | Frequency | Percent | Cumulative Percent |
| 0 Control group | Valid | 1 | 1 | 3,8 | 3,8 |
|  |  | 2 | 5 | 19,2 | 23,1 |
|  |  | 3 | 4 | 15,4 | 38,5 |
|  |  | 4 | 5 | 19,2 | 57,7 |
|  |  | 5 | 6 | 23,1 | 80,8 |
|  |  | 6 | 2 | 7,7 | 88,5 |
|  |  | 7 | 1 | 3,8 | 92,3 |
|  |  | 9 | 1 | 3,8 | 96,2 |
|  |  | Missing | 1 | 3,8 | 100,0 |
|  |  | Total | 26 | 100,0 |  |
| 1 Affected group | Valid | 1 | 5 | 19,2 | 19,2 |
|  |  | 2 | 5 | 19,2 | 38,5 |
|  |  | 3 | 4 | 15,4 | 53,8 |
|  |  | 4 | 2 | 7,7 | 61,5 |
|  |  | 5 | 1 | 3,8 | 65,4 |
|  |  | 6 | 1 | 3,8 | 69,2 |
|  |  | 7 | 1 | 3,8 | 73,1 |
|  |  | 9 | 2 | 7,7 | 80,8 |
|  |  | Missing | 5 | 19,2 | 100,0 |
|  |  | Total | 26 | 100,0 |  |
|  |  |  |  |  |  |
| *FLZ relation to own children (stanine value)* | | | | | |
| Group | | | Frequency | Percent | Cumulative Percent |
| 0 Control group | Valid | 2 | 1 | 3,8 | 3,8 |
|  |  | 3 | 2 | 7,7 | 11,5 |
|  |  | 4 | 4 | 15,4 | 26,9 |
|  |  | 5 | 5 | 19,2 | 46,2 |
|  |  | 6 | 7 | 26,9 | 73,1 |
|  |  | 7 | 5 | 19,2 | 92,3 |
|  |  | 8 | 1 | 3,8 | 96,2 |
|  |  | 9 | 1 | 3,8 | 100,0 |
|  |  | Total | 26 | 100,0 |  |
| 1 Affected group | Valid | 1 | 1 | 3,8 | 4,2 |
|  |  | 3 | 4 | 15,4 | 20,8 |
|  |  | 4 | 4 | 15,4 | 37,5 |
|  |  | 5 | 4 | 15,4 | 54,2 |
|  |  | 6 | 4 | 15,4 | 70,8 |
|  |  | 7 | 5 | 19,2 | 91,7 |
|  |  | 8 | 1 | 3,8 | 95,8 |
|  |  | 9 | 1 | 3,8 | 100,0 |
|  |  | Total | 24 | 92,3 |  |
|  |  | Missing | 2 | 7,7 |  |
|  | Total | | 26 | 100,0 |  |
|  |  |  |  |  |  |
| *FlZ own person (stanine value)* | | | | | |
| Group | | | Frequency | Percent | Cumulative Percent |
| 0 Control group | Valid | 1 | 2 | 7,7 | 7,7 |
|  |  | 2 | 2 | 7,7 | 15,4 |
|  |  | 3 | 3 | 11,5 | 26,9 |
|  |  | 4 | 7 | 26,9 | 53,8 |
|  |  | 5 | 8 | 30,8 | 84,6 |
|  |  | 6 | 3 | 11,5 | 96,2 |
|  |  | 7 | 1 | 3,8 | 100,0 |
|  |  | Total | 26 | 100,0 |  |
| 1 Affected group | Valid | 1 | 4 | 15,4 | 15,4 |
|  |  | 2 | 1 | 3,8 | 19,2 |
|  |  | 3 | 3 | 11,5 | 30,8 |
|  |  | 4 | 3 | 11,5 | 42,3 |
|  |  | 5 | 7 | 26,9 | 69,2 |
|  |  | 6 | 4 | 15,4 | 84,6 |
|  |  | 7 | 1 | 3,8 | 88,5 |
|  |  | 8 | 3 | 11,5 | 100,0 |
|  |  | Total | 26 | 100,0 |  |
|  |  |  |  |  |  |
| *FLZ sexuality (stanine value)* | | | | | |
| Group | | | Frequency | Percent | Cumulative Percent |
| 0 Control group | Valid | 1 | 2 | 7,7 | 7,7 |
|  |  | 2 | 6 | 23,1 | 30,8 |
|  |  | 3 | 4 | 15,4 | 46,2 |
|  |  | 4 | 2 | 7,7 | 53,8 |
|  |  | 5 | 4 | 15,4 | 69,2 |
|  |  | 6 | 3 | 11,5 | 80,8 |
|  |  | 7 | 2 | 7,7 | 88,5 |
|  |  | 8 | 2 | 7,7 | 96,2 |
|  |  | Missing | 1 | 3,8 | 100,0 |
|  |  | Total | 26 | 100,0 |  |
| 1 Affected group | Valid | 1 | 5 | 19,2 | 19,2 |
|  |  | 2 | 4 | 15,4 | 34,6 |
|  |  | 3 | 4 | 15,4 | 50,0 |
|  |  | 4 | 4 | 15,4 | 65,4 |
|  |  | 5 | 1 | 3,8 | 69,2 |
|  |  | 6 | 5 | 19,2 | 88,5 |
|  |  | 7 | 1 | 3,8 | 92,3 |
|  |  | 9 | 1 | 3,8 | 96,2 |
|  |  | Missing | 1 | 3,8 | 100,0 |
|  |  | Total | 26 | 100,0 |  |
|  |  |  |  |  |  |
| *FLZ Friends, relatives (stanine value)* | | | | | |
| Group | | | Frequency | Percent | Cumulative Percent |
| 0 Control group | Valid | 1 | 1 | 3,8 | 3,8 |
|  |  | 2 | 1 | 3,8 | 7,7 |
|  |  | 3 | 4 | 15,4 | 23,1 |
|  |  | 4 | 5 | 19,2 | 42,3 |
|  |  | 5 | 4 | 15,4 | 57,7 |
|  |  | 6 | 2 | 7,7 | 65,4 |
|  |  | 7 | 4 | 15,4 | 80,8 |
|  |  | 8 | 2 | 7,7 | 88,5 |
|  |  | 9 | 2 | 7,7 | 96,2 |
|  |  | Mssing | 1 | 3,8 | 100,0 |
|  |  | Total | 26 | 100,0 |  |
| 1 Affected group | Valid | 1 | 5 | 19,2 | 19,2 |
|  |  | 2 | 3 | 11,5 | 30,8 |
|  |  | 3 | 6 | 23,1 | 53,8 |
|  |  | 4 | 1 | 3,8 | 57,7 |
|  |  | 5 | 5 | 19,2 | 76,9 |
|  |  | 6 | 3 | 11,5 | 88,5 |
|  |  | 7 | 2 | 7,7 | 96,2 |
|  |  | 8 | 1 | 3,8 | 100,0 |
|  |  | Total | 26 | 100,0 |  |
|  |  |  |  |  |  |
| *FLZ apartment (stanine value)* | | | | | |
| Group | | | Frequency | Percent | Cumulative Percent |
| 0 Control group | Valid | 2 | 1 | 3,8 | 3,8 |
|  |  | 3 | 5 | 19,2 | 23,1 |
|  |  | 4 | 4 | 15,4 | 38,5 |
|  |  | 5 | 8 | 30,8 | 69,2 |
|  |  | 6 | 4 | 15,4 | 84,6 |
|  |  | 7 | 2 | 7,7 | 92,3 |
|  |  | 8 | 2 | 7,7 | 100,0 |
|  |  | Total | 26 | 100,0 |  |
| 1 Affected group | Valid | 3 | 3 | 11,5 | 11,5 |
|  |  | 4 | 4 | 15,4 | 26,9 |
|  |  | 5 | 7 | 26,9 | 53,8 |
|  |  | 6 | 7 | 26,9 | 80,8 |
|  |  | 7 | 3 | 11,5 | 92,3 |
|  |  | 8 | 1 | 3,8 | 96,2 |
|  |  | 9 | 1 | 3,8 | 100,0 |
|  |  | Total | 26 | 100,0 |  |
|  |  |  |  |  |  |
| *Family situation* | | | | | |
| Group | | | Frequency | Percent | Cumulative Percent |
| 0 Control group | Valid | married | 20 | 76,9 | 76,9 |
|  |  | single | 1 | 3,8 | 80,8 |
|  |  | divorced | 5 | 19,2 | 100,0 |
|  |  | Total | 26 | 100,0 |  |
| 1 Affected group | Valid | married | 16 | 61,5 | 61,5 |
|  |  | single | 1 | 3,8 | 65,4 |
|  |  | divorced | 9 | 34,6 | 100,0 |
|  |  | Total | 26 | 100,0 |  |
|  |  |  |  |  |  |
| *Occupational status* | | | | | |
| Group | | | Frequency | Percent | Cumulative Percent |
| 0 Control group | Valid | none | 2 | 7,7 | 7,7 |
|  |  | Freelance | 1 | 3,8 | 11,5 |
|  |  | Employee high level | 6 | 23,1 | 34,6 |
|  |  | Employee middle/low level | 7 | 26,9 | 61,5 |
|  |  | Official high level | 1 | 3,8 | 65,4 |
|  |  | Official middle/low level | 3 | 11,5 | 76,9 |
|  |  | Other | 5 | 19,2 | 96,2 |
|  |  | Worker | 1 | 3,8 | 100,0 |
|  |  | Total | 26 | 100,0 |  |
| 1 Affected group | Valid | none | 4 | 15,4 | 15,4 |
|  |  | Freelance | 4 | 15,4 | 30,8 |
|  |  | Self-employed | 1 | 3,8 | 34,6 |
|  |  | Employee middle/low level | 6 | 23,1 | 57,7 |
|  |  | Official high level | 2 | 7,7 | 65,4 |
|  |  | Official middle/low level | 1 | 3,8 | 69,2 |
|  |  | Worker | 2 | 7,7 | 76,9 |
|  |  | Other | 6 | 23,1 | 100,0 |
|  |  | Total | 26 | 100,0 |  |
|  |  |  |  |  |  |
| *Gender* | | | | | |
| Group | | | Frequency | Percent | Cumulative Percent |
| 0 Control group | Valid | female | 23 | 88,5 | 88,5 |
|  |  | male | 3 | 11,5 | 100,0 |
|  |  | Total | 26 | 100,0 |  |
| 1 Affected group | Valid | female | 25 | 96,2 | 96,2 |
|  |  | male | 1 | 3,8 | 100,0 |
|  |  | Total | 26 | 100,0 |  |
|  |  |  |  |  |  |
| *Living alone/together* | | | | | |
| Group | | | Frequency | Percent | Cumulative Percent |
| 0 Control group | Valid | alone | 4 | 15,4 | 15,4 |
|  |  | together with partner | 22 | 84,6 | 100,0 |
|  |  | Total | 26 | 100,0 |  |
| 1 Affected group | Valid | alone | 9 | 34,6 | 34,6 |
|  |  | together with partner | 17 | 65,4 | 100,0 |
|  |  | Total | 26 | 100,0 |  |
|  |  |  |  |  |  |
| *Employed* | | | | | |
| Group | | | Frequency | Percent | Cumulative Percent |
| 0 Control group | Valid | yes | 19 | 73,1 | 73,1 |
|  |  | housewife | 5 | 19,2 | 92,3 |
|  |  | unemployed | 1 | 3,8 | 96,2 |
|  |  | student | 1 | 3,8 | 100,0 |
|  |  | Total | 26 | 100,0 |  |
| 1 Affected group | Valid | Missing | 1 | 3,8 | 3,8 |
|  |  | yes | 12 | 46,2 | 50,0 |
|  |  | own business | 1 | 3,8 | 53,8 |
|  |  | housewife | 12 | 46,2 | 100,0 |
|  |  | Total | 26 | 100,0 |  |
|  |  |  |  |  |  |
| *FLZ total life satisfaction (stanine values)* | | | | | |
| Group | | | Frequency | Percent | Cumulative Percent |
| 0 Control group | Valid | 1 | 2 | 7,7 | 7,7 |
|  |  | 2 | 2 | 7,7 | 15,4 |
|  |  | 3 | 3 | 11,5 | 26,9 |
|  |  | 4 | 7 | 26,9 | 53,8 |
|  |  | 5 | 6 | 23,1 | 76,9 |
|  |  | 6 | 5 | 19,2 | 96,2 |
|  |  | 7 | 1 | 3,8 | 100,0 |
|  |  | Total | 26 | 100,0 |  |
| 1 Affected group | Valid | 1 | 7 | 26,9 | 26,9 |
|  |  | 2 | 4 | 15,4 | 42,3 |
|  |  | 3 | 3 | 11,5 | 53,8 |
|  |  | 4 | 5 | 19,2 | 73,1 |
|  |  | 5 | 4 | 15,4 | 88,5 |
|  |  | 6 | 3 | 11,5 | 100,0 |
|  |  | Total | 26 | 100,0 |  |
|  |  |  |  |  |  |
| *FLZ total life satisfaction (raw values)* | | | | | |
| Group | | | Frequency | Percent | Cumulative Percent |
| 0 Control group | Valid | 159 | 1 | 3,8 | 3,8 |
|  |  | 167 | 1 | 3,8 | 7,7 |
|  |  | 192 | 1 | 3,8 | 11,5 |
|  |  | 207 | 1 | 3,8 | 15,4 |
|  |  | 219 | 1 | 3,8 | 19,2 |
|  |  | 222 | 1 | 3,8 | 23,1 |
|  |  | 235 | 1 | 3,8 | 26,9 |
|  |  | 237 | 1 | 3,8 | 30,8 |
|  |  | 241 | 1 | 3,8 | 34,6 |
|  |  | 242 | 1 | 3,8 | 38,5 |
|  |  | 246 | 1 | 3,8 | 42,3 |
|  |  | 250 | 2 | 7,7 | 50,0 |
|  |  | 251 | 1 | 3,8 | 53,8 |
|  |  | 255 | 1 | 3,8 | 57,7 |
|  |  | 257 | 1 | 3,8 | 61,5 |
|  |  | 258 | 1 | 3,8 | 65,4 |
|  |  | 261 | 1 | 3,8 | 69,2 |
|  |  | 263 | 1 | 3,8 | 73,1 |
|  |  | 271 | 1 | 3,8 | 76,9 |
|  |  | 273 | 1 | 3,8 | 80,8 |
|  |  | 274 | 1 | 3,8 | 84,6 |
|  |  | 278 | 1 | 3,8 | 88,5 |
|  |  | 281 | 1 | 3,8 | 92,3 |
|  |  | 282 | 1 | 3,8 | 96,2 |
|  |  | 291 | 1 | 3,8 | 100,0 |
|  |  | Total | 26 | 100,0 |  |
| 1 Affected group | Valid | 132 | 1 | 3,8 | 3,8 |
|  |  | 156 | 1 | 3,8 | 7,7 |
|  |  | 165 | 1 | 3,8 | 11,5 |
|  |  | 167 | 1 | 3,8 | 15,4 |
|  |  | 169 | 1 | 3,8 | 19,2 |
|  |  | 173 | 1 | 3,8 | 23,1 |
|  |  | 181 | 1 | 3,8 | 26,9 |
|  |  | 191 | 1 | 3,8 | 30,8 |
|  |  | 199 | 2 | 7,7 | 38,5 |
|  |  | 204 | 1 | 3,8 | 42,3 |
|  |  | 215 | 2 | 7,7 | 50,0 |
|  |  | 233 | 1 | 3,8 | 53,8 |
|  |  | 241 | 1 | 3,8 | 57,7 |
|  |  | 244 | 1 | 3,8 | 61,5 |
|  |  | 249 | 2 | 7,7 | 69,2 |
|  |  | 253 | 1 | 3,8 | 73,1 |
|  |  | 255 | 1 | 3,8 | 76,9 |
|  |  | 260 | 1 | 3,8 | 80,8 |
|  |  | 269 | 2 | 7,7 | 88,5 |
|  |  | 277 | 1 | 3,8 | 92,3 |
|  |  | 283 | 1 | 3,8 | 96,2 |
|  |  | 284 | 1 | 3,8 | 100,0 |
|  |  | Total | 26 | 100,0 |  |
|  |  |  |  |  |  |
| *FLZ dichotomous* | | | | | |
| Group | | | Frequency | Percent | Cumulative Percent |
| 0 Control group | Valid | “bad” (FLZ<3) | 7 | 26,9 | 26,9 |
|  |  | “good” (FLZ>3) | 19 | 73,1 | 100,0 |
|  |  | Total | 26 | 100,0 |  |
| 1 Affected group | Valid | “bad” (FLZ<3) | 14 | 53,8 | 53,8 |
|  |  | “good” (FLZ>3) | 12 | 46,2 | 100,0 |
|  |  | Total | 26 | 100,0 |  |
|  |  |  |  |  |  |
|  |  |  |  |  |  |
| *Support during daytime dichotomous* | | | | | |
| Group | | | Frequency | Percent | Cumulative Percent |
| 0 Control group | Missing | System | 26 | 100,0 |  |
| 1 Affected group | Valid | sufficient | 14 | 53,8 | 53,8 |
|  |  | insufficient | 12 | 46,2 | 100,0 |
|  |  | Total | 26 | 100,0 |  |
|  |  |  |  |  |  |
| *Support during night time dichotomous* | | | | | |
| Group | | | Frequency | Percent | Cumulative Percent |
| 0 Control group | Missing | System | 26 | 100,0 |  |
| 1 Affected group | Valid | sufficient | 20 | 76,9 | 76,9 |
|  |  | insufficient | 6 | 23,1 | 100,0 |
|  |  | Total | 26 | 100,0 |  |
|  |  |  |  |  |  |
| *Home visits dichotomous* | | | | | |
| Group | | | Frequency | Percent | Cumulative Percent |
| 0 Control group | Missing | System | 26 | 100,0 |  |
| 1 Affected group | Valid | sufficient | 9 | 34,6 | 34,6 |
|  |  | insufficient | 17 | 65,4 | 100,0 |
|  |  | Total | 26 | 100,0 |  |
|  |  |  |  |  |  |
| *Emergencies* | | | | | |
| Group | | | Frequency | Percent | Cumulative Percent |
| 0 Control group | Missing | System | 26 | 100,0 |  |
| 1 Affected group | Valid | few | 15 | 57,7 | 57,7 |
|  |  | many | 11 | 42,3 | 100,0 |
|  |  | Total | 26 | 100,0 |  |
|  |  |  |  |  |  |
| *Stress general* | | | | | |
| Group | | | Frequency | Percent | Cumulative Percent |
| 0 Control group | Missing | System | 26 | 100,0 |  |
| 1 Affected group | Valid | 4 | 4 | 15,4 | 15,4 |
|  |  | 5 | 5 | 19,2 | 34,6 |
|  |  | 6 | 4 | 15,4 | 50,0 |
|  |  | 7 | 3 | 11,5 | 61,5 |
|  |  | 8 | 5 | 19,2 | 80,8 |
|  |  | 9 | 4 | 15,4 | 96,2 |
|  |  | 10 | 1 | 3,8 | 100,0 |
|  |  | Total | 26 | 100,0 |  |
|  |  |  |  |  |  |
| *Stress dichotomous* | | | | | |
| Group | | | Frequency | Percent | Cumulative Percent |
| 0 Control group | Missing | System | 26 | 100,0 |  |
| 1 Affected group | Valid | low | 14 | 53,8 | 53,8 |
|  |  | high | 12 | 46,2 | 100,0 |
|  |  | Total | 26 | 100,0 |  |
|  |  |  |  |  |  |
| *Age at the time of injury* | | | | | |
| Group | | | Frequency | Percent | Cumulative Percent |
| 0 Control group | Missing | System | 26 | 100,0 |  |
| 1 Affected group | Valid | Early (< 3 y) | 13 | 50,0 | 50,0 |
|  |  | Late (> 3 y) | 13 | 50,0 | 100,0 |
|  |  | Total | 26 | 100,0 |  |
|  |  |  |  |  |  |
| *Index of burden* | | | | | |
| Group | | | Frequency | Percent | Cumulative Percent |
| 0 Control group | Missing | System | 26 | 100,0 |  |
| 1 Affected group | Valid | 1 | 2 | 7,7 | 7,7 |
|  |  | 2 | 1 | 3,8 | 11,5 |
|  |  | 3 | 7 | 26,9 | 38,5 |
|  |  | 4 | 4 | 15,4 | 53,8 |
|  |  | 5 | 7 | 26,9 | 80,8 |
|  |  | 6 | 2 | 7,7 | 88,5 |
|  |  | 7 | 3 | 11,5 | 100,0 |
|  |  | Total | 26 | 100,0 |  |
|  |  |  |  |  |  |
| *FFCv Index dichotomous* | | | | | |
| Group | | | Frequency | Percent | Cumulative Percent |
| 0 Control group | Missing | System | 26 | 100,0 |  |
| 1 Affected group | Valid | 1 | 15 | 57,7 | 57,7 |
|  |  | 2 | 11 | 42,3 | 100,0 |
|  |  | Total | 26 | 100,0 |  |
