# Supplementary material for: Life satisfaction in families with a child in an Unresponsive Wakefulness Syndrome
Source: BMC Pediatr. 2021 Mar 8;21:116. doi: 10.1186/s12887-021-02549-8 (PMC7938537; doi:10.1186/s12887-021-02549-8)
Supplement: Supplementary file 3 — Additional file 3. Supplement 3. Table III. Results of the ANOVA for overall Life Satisfaction. [file 12887_2021_2549_MOESM3_ESM.docx]

Supplement 3

Table III. Results of the ANOVA for overall Life Satisfaction

| **Tests of Between-Subjects Effects** | | | | | | |  |
| --- | --- | --- | --- | --- | --- | --- | --- |
| Dependent variable: Stanine values of Life-satisfaction (total score**)** | | | | | | |  |
| **Source** | Type III Sum of Squares | df | Mean Square | **F** | **p** | **Part. Eta-Quadrat** | **Parameter Estimate B** |
| Corrected Model | 42.280 | 3 | 14.093 | 8.36 | .001 | .533 |  |
| Self-management (FERUS) | 7.487 | 1 | 7.487 | 4.44 | .047 | .168 | .080 |
| Stress situation (FFCv) | 18.593 | 1 | 18.593 | 11.02 | .003 | .334 | -.520 |
| Experienced emotions (SEE) | 3.259 | 1 | 3.259 | 1.93 | .178 | .081 | -.045 |
